# Global genome splicing analysis reveals an increased number of alternatively spliced genes with aging

**DOI:** 10.1111/acel.12433

**Published:** 2015-12-21

**Authors:** Sofía A. Rodríguez, Diana Grochová, Tomás McKenna, Bhavesh Borate, Niraj S. Trivedi, Michael R. Erdos, Maria Eriksson

**Affiliations:** ^1^ Department of Biosciences and NutritionCenter for Innovative MedicineKarolinska InstitutetNovumSE‐141 83HuddingeSweden; ^2^ National Human Genome Research InstituteNational Institutes of HealthBethesdaMDUSA

**Keywords:** aging, alternative splicing, HGPS, NF‐κB, progeria, spliceosome, transcriptome

## Abstract

Alternative splicing (AS) is a key regulatory mechanism for the development of different tissues; however, not much is known about changes to alternative splicing during aging. Splicing events may become more frequent and widespread genome‐wide as tissues age and the splicing machinery stringency decreases. Using skin, skeletal muscle, bone, thymus, and white adipose tissue from wild‐type C57BL6/J male mice (4 and 18 months old), we examined the effect of age on splicing by AS analysis of the differential exon usage of the genome. The results identified a considerable number of AS genes in skeletal muscle, thymus, bone, and white adipose tissue between the different age groups (ranging from 27 to 246 AS genes corresponding to 0.3–3.2% of the total number of genes analyzed). For skin, skeletal muscle, and bone, we included a later age group (28 months old) that showed that the number of alternatively spliced genes increased with age in all three tissues (*P *<* *0.01). Analysis of alternatively spliced genes across all tissues by gene ontology and pathway analysis identified 158 genes involved in RNA processing. Additional analysis of AS in a mouse model for the premature aging disease Hutchinson–Gilford progeria syndrome was performed. The results show that expression of the mutant protein, progerin, is associated with an impaired developmental splicing. As progerin accumulates, the number of genes with AS increases compared to in wild‐type skin. Our results indicate the existence of a mechanism for increased AS during aging in several tissues, emphasizing that AS has a more important role in the aging process than previously known.

## Introduction

Aging is a multifactorial process that affects most biological functions and is most likely caused by a mixture of molecular events. Recent advances in the study of global patterns of gene expression with the use of microarrays and bioinformatics have provided new insights into the aging mechanisms of different tissues, organs, and systems (Hsia & Cornwall, 2004; Lazuardi *et al*., 2009). Additionally, the significance of the global diversity of the transcriptome generated by alternative splicing (AS) and its contribution to different biological processes has begun to be investigated in more detail only recently. AS is thought to have several roles in complex organisms, primarily in increasing transcriptome diversity (Chen & Manley, 2009). Previously thought to be a relatively uncommon phenomenon, AS has recently been shown to be widespread throughout the genome (Modrek & Lee, 2002). Studies in rats and mice have shown that increased age is associated with changes in mRNA processing leading to aberrant splicing (Yannarell *et al*., 1977; Meshorer & Soreq, 2002). Normal aging has also been found to be associated with an aberrant increase in the production and maturation of many mRNAs in human peripheral blood leukocytes (Harries *et al*., 2011). Studies with human blood, endothelial cells, and fibroblasts also showed that splicing factor genes had a reproducible expression change with age (Holly *et al*., 2013). There are different types of AS (Black & Grabowski, 2003) that normally occur in a regulated manner, but aberrant splicing is commonly associated with disease. More than 15% of heritable human diseases are known to be associated with mutations in splice sites or splicing regulatory elements (Maniatis & Tasic, 2002).

Studies of genes and molecular processes that are associated with segmental progeroid disorders, such as Hutchinson–Gilford progeria syndrome (HGPS, progeria, OMIM#176670), could be of importance when studying the genetic mechanisms of aging (Martin, 2005; Baker *et al*., 1981). For example, most cases of HGPS are caused by a *de novo* point mutation in the *LMNA* gene (*LMNA* c.1824C>T; p.G608G). This mutation activates a cryptic splice site that results in aberrant splicing of the lamin A transcript (Eriksson *et al*., 2003). Interestingly, it has been shown that the products of this aberrant splicing, the truncated transcript and resultant protein (named progerin), increase in number with aging in HGPS (Goldman *et al*., 2004; Cao *et al*., 2007; Rodriguez *et al*., 2009). In addition, several reports have found progerin, and increasing levels of progerin, in normal cells over the course of normal aging (Scaffidi & Misteli, 2006; McClintock *et al*., 2007; Cao *et al*., 2007; Rodriguez *et al*., 2009), which suggests a similar genetic mechanism in HGPS and normal aging. Moreover, genome‐scale expression profiling in cells from HGPS patients, as well as in physiological aging, has revealed widespread transcriptional misregulation in multiple mammalian tissues (Ly *et al*., 2000; Csoka *et al*., 2004; Zahn *et al*., 2007; Scaffidi & Misteli, 2008; Cao *et al*., 2011; McCord *et al*., 2013).

In this study, we wanted to analyze whether the noted increase in progerin transcripts that occurs with cellular and tissue aging (in HGPS and physiological aging) could be the result of a more general aging effect that would be evident by a global increase in alternative exon usage with aging. In addition, we wondered whether increased alternative exon usage could be caused by a decreased stringency and/or efficiency of the RNA splicing machinery, possibly as a function of increased age. To try to study these processes, we have hybridized exon expression microarrays with single‐stranded cDNA derived from the RNA of five different tissues (skin, bone, skeletal muscle, white adipose tissue, and thymus), from 4‐ and 18‐month‐old wild‐type mice. To investigate age‐related changes, cDNA from the skin, bone, and skeletal muscle from an additional age group of 28‐month‐old wild‐type male mice was also analyzed. In addition, cDNA from keratinocytes from a mouse model with induced expression of the most common HGPS mutation was also hybridized to exon arrays. The effect of age on splicing was investigated by querying the expression level of exons and using Partek's GS alternative splicing ANOVA to determine the number of AS genes between different age groups.

## Results

### Age‐related degeneration in skeletal muscle and skin

**Figure 1:**
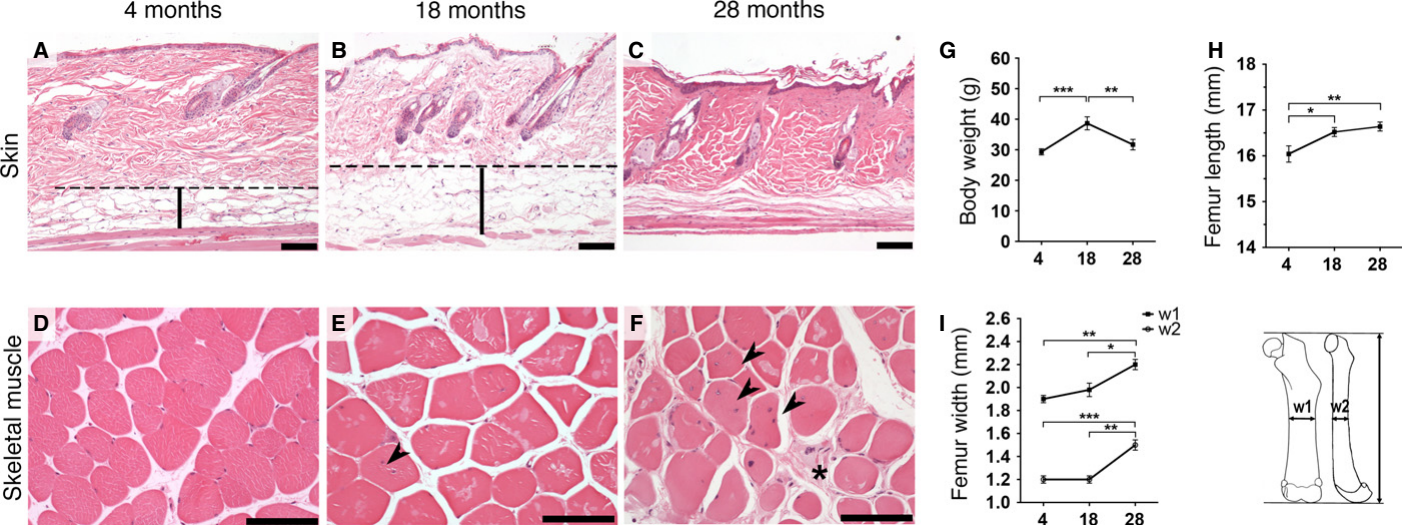
Age‐related degenerative changes in wild‐type mice. Representative hematoxylin and eosin staining of skin (A–C) and skeletal muscle (D–F). (A–B) Increased initial thickening of the hypodermal fat layer in 18‐month‐old mice (B) becomes virtually absent in 28‐month‐old mice with terminal marked dermal fibrosis (C). (D–F) Increased degenerative changes with age in muscle. (D) 4‐month‐old mice showed essentially no degenerative changes. (E) 18‐month‐old mice have mild variation in fiber size and rare centralization of the nuclei (arrowhead). (F) 28‐month‐old animals present marked variable fiber size, frequent nuclear centralization (arrowheads), and endomysial fibrosis (star). (G) A significant increase in body weight between the 4‐ and 18‐month‐old mice decreased at 28 months. (H) Femur lengths with initial increase and posterior plateau phase. (I) Femur thickness measured in the mid‐diaphysis of 2 bone dimensions (w1 and w2). Dashed line (hypodermal mean upper limit). Vertical bars (hypodermis). Scale bars 100 μm. *P <* *0.05, **P <* *0.01 and ***P <* *0.001; one‐tailed paired *t*‐test, the data are expressed as the means ± SEM.

To analyze the differences in cellular composition in tissues at different ages and to confirm an expected age‐related pathology, samples from the same animals and tissues (skeletal muscle and skin) used for exon arrays were sectioned and analyzed for histopathology. Histological examination of hematoxylin and eosin‐stained sections from the skin and skeletal muscle showed expected age‐related degeneration features in both skin (Fig. 1A–C) and skeletal muscle (Fig. 1D–F) consistent with normal physiological aging. The hypodermal fat layer showed an initial increase in the 18‐month‐old mice (Fig. 1B), followed by a virtually complete loss in the 28‐month‐old mice together with marked dermal fibrosis (Fig. 1C). Skeletal muscle showed gradual degenerative changes including variable muscle fiber diameter, muscle fiber splitting, fiber fragmentation and dissolution, satellite cell activation, endomysial fibrosis, and mild to moderate regeneration (centralization of nuclei), which was prominent in the 28‐month‐old mice (Fig. 1F). Body weights showed an initial body weight gain (from 4 to 18 months) and subsequent body weight loss in the later aging period (from 18 to 28 months) (Fig. 1G). Femur measurements showed age‐related growth that was consistent with that of previous reports (Fig. 1H) (Glatt *et al*., 2007; Sohal *et al*., 2009). Femur mid‐diaphysis thickness was consistent with increased periosteal perimeter (Fig. 1I) (Ferguson *et al*., 2003). Histopathological changes were more significant between 4‐ and 28‐month‐old animals, compared to the changes observed between 4‐ and 18‐month‐old animals, for both the skeletal muscle and skin.

### The number of alternatively spliced genes increases during physiological aging

**Figure 2:**
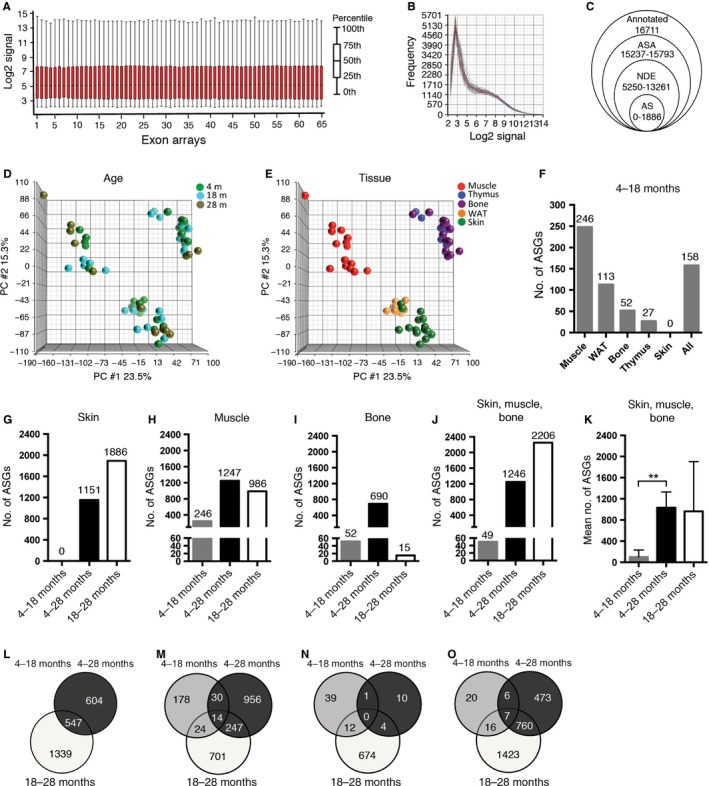
Alternative splicing analysis showed an increased number of alternatively spliced genes during aging. (A) Box‐plot overview of all probe set log2 expression values after normalization and summarization. (B) Distribution of log2 signal frequencies shows homogenous distribution across all 65 exon arrays. (C) Gene filtering for AS analysis in Partek: 194 293 probe sets were summarized in a total of 16 711 annotated genes. Fewer genes were considered by the alternative splicing analysis ANOVA, after the removal of DE genes (unadjusted age *P*‐value <0.05) and non‐DE genes (NDE). AS genes with FDR‐adjusted alt‐splice *P*‐value <0.05 were considered to be AS and were used in this study. (D–E) Principal component analysis (PCA): mapping of all 65 exon arrays grouped by age (D) and tissue (E) shows that the main variability is observed between different tissues and not according to age. (F) AS analysis between 4‐ and 18‐month‐old animals shows, with the exception of skin, a considerable number of AS genes (ASGs) in each tissue and across all tissues. (G–J) ASGs between all 3 age groups in skin (G), muscle (H), bone (I), across tissues (J) and mean number of ASGs ± SEM in the skin, muscle, and bone showed significantly more differences in ASGs between 4‐ and 28‐month‐old animals than between 4‐ and 18‐month‐old animals (K). (L–O) Venn diagrams of overlapping ASGs between different age groups in the skin (L), muscle (M), bone (N) and across skin, muscle, and bone (O) showed a considerable amount of both overlap and pairwise comparison‐specific ASGs between different age groups. WAT, white adipose tissue. **P <* *0.01: two‐tailed, unpaired *t*‐test, the data are expressed as the means ± SEM.

We used exon expression microarrays to investigate alternative exon usage in RNA from five different tissues and two or three ages of wild‐type mice. RNA samples (*N* = 5 per age group and tissue, 65 samples in total) were labeled and hybridized to mouse exon arrays. The Partek quality check showed a homogeneous distribution of probe set expression values and signal frequencies across the 65 exon arrays (Fig. 2A,B). AS analysis and gene filtering reduced the number of genes from 16 711 well‐annotated genes to a range of 0–1886 AS genes, depending on the tissue and age groups compared (Fig. 2C and File S1). This corresponded to a range of 0–30.4% of the total amount of genes that were analyzed (File S1). Principal component analysis (PCA) for the multivariate data in the 65 arrays revealed that the majority of the differences between arrays were not grouped by age (Fig. 2D), but instead represented differences between tissues (Fig. 2E). AS analysis revealed a considerable number of AS genes in all 5 tissues and between most age groups (Fig. 2F–K). Analysis of the AS genes between the 4‐ and 18‐month‐old age groups showed that AS genes were found in most tissues; AS analysis across all 5 tissues yielded a total of 158 genes (Fig. 2F). Surprisingly, there were no AS genes found between 4‐ and 18‐month‐old skin specimens (Fig. 2F,G). In contrast, the skin tissue of 18‐ and 28‐month‐old animals showed the highest number of AS genes (1886 genes, corresponding to 30.4% of the total number of genes analyzed) (Fig. 2G and File S1). The tissue that showed the second‐lowest number, 15 genes (corresponding to 0.2% of the total number of genes analyzed), was the bone in the pairwise comparison between 18‐ and 28‐month‐old mice (Fig. 2I and File S1).

To determine whether increasing numbers of alternatively spliced genes correlated with increasing age, we compared the number of AS genes obtained in the 4‐ to 18‐month period to those obtained in the 4‐ to 28‐month age period. The results showed that the number of AS genes in the 4‐ to 28‐month age period (690–1247 genes, corresponding to 13.1–19.1% of the total number of the genes analyzed) was higher than in the 4‐ to 18‐month period (0–246 genes, corresponding to 0–3.2% of the total number of the genes analyzed) for the three tissues analyzed (skin, skeletal muscle, and bone) (Fig. 2G–I and File S1). Similar results were obtained when AS analysis was performed across all three tissues (Fig. 2J), indicating that the number of AS genes increased significantly with advanced age in these tissues (*P *<* *0.01, Fig. 2K). However, when comparing the number of AS genes for the period of 4–18 months with the period of 18–28 months, the number of AS genes was higher in the later aging period, 18–28 months, for the skin and muscle (Fig. 2G,H), in contrast to bone, where there were more AS genes in the earlier aging period, 4–18 months (Fig. 2I). There was a partial overlap of genes that were alternatively spliced between all different aging periods, which suggested that each aging period had both period‐specific AS, in addition to AS that occurred over several aging periods (Fig. 2L–O). The AS gene overlap was most pronounced between periods with higher numbers of AS genes, and when analyzed across all tissues, the biggest AS gene overlap was found between the later aging periods, 4–28 months compared with 18–28 months (760 genes, Fig. 2O). However, the 4‐ to 18‐month period had only 49 genes with AS, of which 32.7% or 12.2% of the genes with AS were shared with either the 18‐ to 28‐month or the 4‐ to 28‐month period, respectively (Fig. 2O).

### Gene Ontology, Pathway, and Network analysis implicate age‐related alternative splicing in post‐transcriptional RNA processing and Cancer

**Figure 3:**
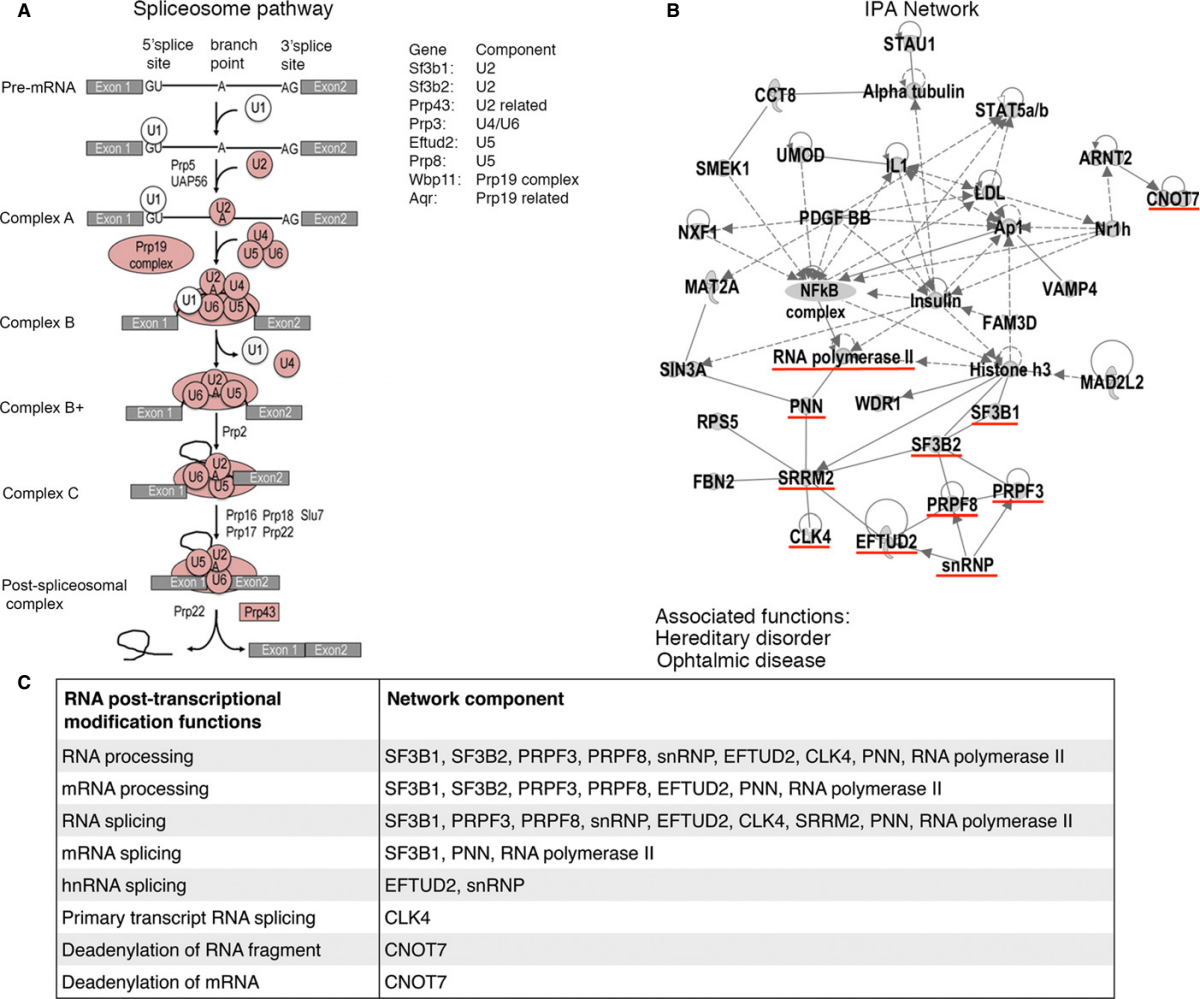
Alternatively spliced genes with age were enriched for post‐transcriptional modifications including the spliceosome pathway. (A) The mouse KEGG spliceosome pathway with affected components highlighted in pink and AS genes for each component. (B) IPA network analysis of AS genes and main associated network functions. Highlighted underlined in red are the AS genes and components involved in RNA post‐transcriptional processes such as splicing. (C) RNA post‐transcriptional modification functions and the corresponding network components and genes that were identified as being alternatively spliced in aging.

Biological functions and relationships of the AS genes obtained for the earlier aging period (4–18 months) were analyzed across the 5 tissues. A gene set of 158 AS genes (Fig. 2F, File S1) was analyzed with webgestalt (Zhang *et al*., 2005; Wang *et al*., 2013) and IPA (Ingenuity^®^ Systems, www.ingenuity.com). The most significant gene ontology (GO) biological processes enriched with AS genes were macromolecule metabolic processing and its subcategory RNA processing, including functions such as mRNA processing and RNA splicing (Table 1). Interestingly, within the enriched cellular components (Table S1), the most significant location was the nuclei and AS gene products were also confined within the spliceosomal complex and specifically within the catalytic step 2 of the spliceosome (Table S1). These results were further confirmed when evaluating enriched KEGG pathways, where the spliceosome was the most significantly enriched pathway (Table 1, Fig. 3A). The most significant results from the IPA enrichment analysis for the canonical pathway category corresponded to the EIF2 signaling pathway, and for the molecular and cellular functions category, the RNA post‐transcriptional modification function (Table 1, Fig. 3B, 3C). Cancer was the most significant among the diseases and disorders category with 124 AS genes corresponding to 78.5% of the 158 AS genes on the list (Table 1). In summary, RNA post‐transcriptional modifications such as RNA processing and RNA splicing were functions consistently obtained in the different enrichment and pathway analyses performed (Table 1; GO, KEGG and IPA). In addition, the RNA post‐transcriptional modification network‐associated function was included in the second‐highest‐scored IPA network (Table 1, Fig. 3B), and associated functions to the network components therein were mainly RNA processing, mRNA processing, RNA splicing, and mRNA splicing (Fig. 3C). Finally, this network revealed several gene relationships converging into a major NF‐kB complex node (Fig. 3B).

**Table 1:** Enrichment analysis of alternative spliced genes between 4‐ and 18‐month‐old animals across five tissues

Category	*P* value/score	No. of genes	% of genes on list^a^
GO biological processes^WG^	*P* value		
Macromolecule metabolic process	1.00E‐04	77	48.7
Cellular macromolecule metabolic process	1.00E‐04	72	45.6
RNA processing	1.00E‐04	17	10.8
mRNA processing	1.00E‐04	13	8.2
RNA splicing	3.00E‐04	11	7.0
KEGG pathways^WG^	*P* value		
Spliceosome	1.00E‐04	8	5.1
Cell cycle	3.96E‐02	5	3.2
mRNA surveillance pathway	3.96E‐02	4	2.5
RNA transport	4.78E‐02	5	3.2
Protein processing in ER	5.45E‐02	5	3.2
Canonical pathways^IPA^	*P* value		
EIF2 signaling	1.44E‐02	5	3.2
ATM signaling	1.51E‐02	3	1.9
GDP‐L‐fucose biosynthesis II (from L‐fucose)	1.78E‐02	1	0.6
Protein ubiquitination pathway	2.15E‐02	6	3.8
Estrogen receptor signaling	2.44E‐02	4	2.5
Molecular and cellular functions^IPA^	*P* value		
RNA post‐transcriptional modification	1.55E‐04 – 4.78E‐02	11	7.0
Cellular development	2.29E‐04 – 4.91E‐02	14	8.9
Protein synthesis	9.87E‐04 – 3.52E‐02	15	9.5
Cellular assembly and organization	1.08E‐03 – 4.38E‐02	20	12.7
Cell cycle	1.74E‐03 – 4.38E‐02	13	8.2
Diseases and disorders^IPA^	*P* value		
Cancer	1.55E‐09 – 4.78E‐02	124	78.5
Gastrointestinal disease	1.55E‐09 – 4.08E‐02	73	46.2
Organismal injury and abnormalities	1.61E‐07 – 4.38E‐02	75	47.5
Reproductive system	1.61E‐07 – 4.38E‐02	59	37.3
Infectious disease	1.74E‐03 – 4.38E‐02	13	8.2
Networks^IPA^ and network‐associated functions	Score		
Cellular assembly and organization, developmental disorder, hereditary disorder	48	24	15.2
RNA post‐transcriptional modification; hereditary disorder, ophthalmic disease	46	23	14.6
Organ morphology, reproductive system development and function, embryonic development	38	20	12.7
Cancer, gastrointestinal disease, inflammatory disease	31	18	11.4
Cell signaling, post‐translational modification, protein synthesis	28	16	10.1

a Number of genes on list = 158, ^WG^Enrichment analysis performed with WebGestalt, – Subcategory, ^IPA^Enrichment analysis performed with ingenuity pathway analysis.

### The number of alternatively spliced genes increases with age in a HGPS mouse model

**Figure 4:**
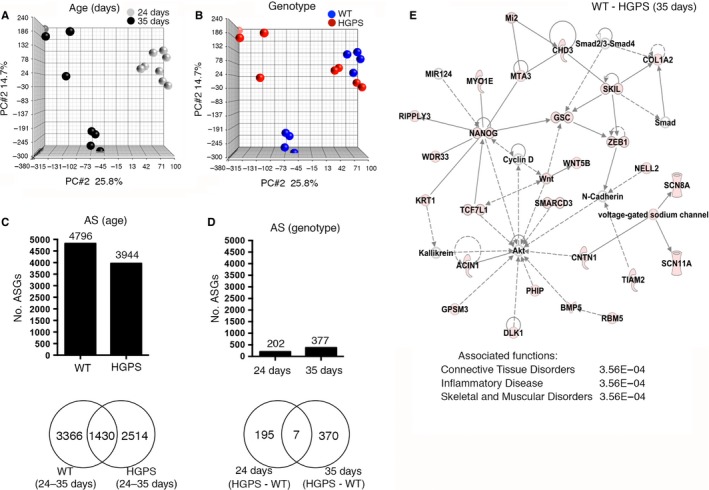
Expression of the HGPS mutation impairs development‐specific alternative splicing. (A–B) PCA mapping of keratinocyte exon arrays from HGPS mice and their wild‐type littermate controls shows that no major differences are observed after the expression of progerin for 24 days. The main difference is observed between 24‐ and 35‐day‐old animals (A) and secondarily between wild‐type and HGPS mice at 35 days of age (B). (C) The number of AS genes with an FDR‐adjusted alt‐splice *P*‐value <0.05 in wild‐type and HGPS mice during the 24‐ to 35‐day developmental period (age) is reduced upon expression of the HGPS mutation. (D) The number of AS genes between wild‐type and HGPS is higher at postnatal day 35 compared to postnatal day 24. (E) IPA network and main associated network functions of 377 AS genes between 35‐day‐old HGPS mice and their wild‐type littermates.

To investigate whether an increased number of AS genes were also observed in HGPS, a disorder characterized by pathological premature aging, we analyzed keratinocytes from a previously generated HGPS mouse model (Sagelius *et al*., 2008a). This model has inducible expression of the most common HGPS mutation, *LMNA* c.1824C>T; p.G608G, under the regulation of the keratin 5 promotor with expression in basal keratinocytes of the interfollicular epidermis (Sagelius *et al*., 2008a). Two age periods (postnatal days 24 and 35) were chosen because at postnatal day 24 the skin phenotype is not apparent in contrast to postnatal day 35, which shows a quite severe skin disease (Sagelius *et al*., 2008b). These time points were also chosen because they represent different stages of the hair cycle (Hanif *et al*., 2009). PCA mapping of hybridization data analyzed for AS using the partek software revealed that the main difference in the keratinocyte data sets was observed between the 24‐ and 35‐day‐old wild‐type mice (Fig. 4A) and secondarily between the 35‐day‐old HGPS mice and their wild‐type littermates (Fig. 4B). The PCA plot revealed that at postnatal day 24, there was no clear separation between the HGPS and wild‐type littermates (Fig. 4A,B). However, at postnatal day 35, the data sets from HGPS and wild‐type littermates had separated in different populations (Fig. 4A,B). The number of AS genes in the wild‐type between postnatal days 24 and 35 was 4796 genes (corresponding to 64.6% of the total number of genes analyzed) (Fig. 4C and File S2). Similarly, analysis of AS genes in HGPS showed that 3944 genes were AS between postnatal days 24 and 35 (corresponding to 39.4% of the total number of genes analyzed) (Fig. 4C and File S2). This lower number of alternatively spliced genes, in HGPS relative to wild‐type with increased age, suggested that expression of the HGPS mutation reduced developmentally regulated splicing. The number of alternatively spliced genes shared between the wild‐type and HGPS was 1430, which corresponded to 19.6% of the total number of alternatively spliced genes (Fig. 4C). The number of AS genes shared between wild‐type and HGPS was higher at 35 days (377 genes, corresponding to 4.1% of the total number of genes analyzed) compared to 24 days (202 genes, corresponding to 1.5% of the total number of genes analyzed) (Fig. 4D, File S2). Seven AS genes were shared between the 24‐ and 35‐day time points, which corresponded to 1.2% of the total number of alternatively spliced genes (Fig. 4D). Taken together, the results showed that initial expression of the HGPS mutation was associated with a reduced degree of developmental‐regulated splicing, but that the accumulation of progerin and progression of the disease showed an association with increased number of AS genes between HGPS and wild‐type.

### GO, Pathway, and Network analysis of age‐related alternatively spliced genes in HGPS

The 24‐day‐old HGPS and wild‐type mice did not map as separate groups in the PCA (Fig. 4A,B). The external skin phenotype in the HGPS mouse was only starting to become visible at postnatal week 4, and the development of the disease phenotype was more pronounced at 35 days of postnatal transgenic expression (Sagelius *et al*., 2008a,b). We chose to perform gene enrichment analysis on the set of 377 AS genes obtained between the 35‐day‐old HGPS mice and their wild‐type littermates (Fig. 4D, File S2). The most significant GO biological processes enriched with AS genes were response to amino acid stimulus and skin development (Table 2). AS genes were confined to intracellular locations and the fibrillar collagen of the ECM (Table S2).

**Table 2:** Enrichment analysis of alternative spliced genes between keratinocytes from 35‐day‐old HGPS mice and their wild‐type littermates

Category	*P* value/score	No. of genes	% of genes on list^a^
GO biological processes^WG^	Adj. *P* value		
Response to amino acid stimulus	6.00E‐04	8	2.1
Skin development	6.00E‐04	9	2.4
Primary metabolic process	1.40E‐03	181	48.0
Organic substance metabolic process	1.60E‐03	186	49.3
Negative regulation of cell adhesion	1.60E‐03	10	2.7
KEGG pathways^WG^	Adj. *P* value		
Protein digestion and absorption	1.70E‐03	9	2.4
Amebiasis	3.40E‐03	10	2.7
ECM–receptor interaction	6.30E‐03	8	2.1
Focal adhesion	1.19E‐02	12	3.2
Melanogenesis	3.96E‐02	7	1.9
Canonical pathways^IPA^	*P* value		
Intrinsic prothrombin activation pathway	1.51E‐05	6	1.6
Cardiomyocyte differentiation via BMP receptors	6.31E‐03	3	0.8
DNA methylation and transcriptional repression signaling	8.54E‐03	3	0.8
Role of NANOG in mammalian embryonic stem cell pluripotency	9.14E‐03	7	1.9
Histidine degradation II	1.18E‐02	2	0.5
Molecular and cellular functions^IPA^	*P* value		
Cell‐to‐cell signaling and interaction	2.02E‐04 – 2.15E‐02	37	9.8
Cellular assembly and organization	3.23E‐04 – 2.14E‐02	49	13.0
Cell cycle	4.28E‐04 – 2.15E‐02	43	11.4
Cell death and survival	4.60E‐04 – 2.15E‐02	49	13.0
Cellular development	4.60E‐04 – 2.15E‐02	68	18.0
Diseases and disorders^IPA^	*P* value		
Connective tissue disorders	4.47E‐09 – 2.15E‐02	35	9.3
Organismal injury and abnormalities	1.97E‐07 – 2.15E‐02	126	33.4
Dermatological diseases and conditions	2.72E‐07 – 2.15E‐02	40	10.6
Developmental disorder	9.83E‐06 – 2.15E‐02	69	18.3
Hereditary disorder	9.83E‐06 – 2.15E‐02	64	17.0
Networks^IPA^ and associated network functions	Score		
Connective tissue disorders, inflammatory disease, skeletal and muscular disorders	40	25	6.6
Organismal injury and abnormalities, reproductive system disease, nervous system development and function	38	24	6.4
Cell‐mediated immune response, cellular development, cellular function and maintenance	34	22	5.8
Gene expression, RNA post‐transcriptional modification, cancer	32	22	5.8
Protein synthesis, cellular movement, hereditary disorder	26	18	4.8

a Number of genes on list = 377, ^WG^Enrichment analysis performed with WebGestalt, – Subcategory, ^IPA^Enrichment analysis performed with ingenuity pathway analysis.

The results from the IPA enrichment analysis of AS genes between 35‐day‐old HGPS and wild‐type mice showed several overlaps with the results obtained for the most differently aged data set to identify AS in the physiological aging of skin, that is, the AS genes in the 4‐ to 28‐month age period of the skin (compare Table 2 and Table S3). Shared top‐5 functions within the IPA molecular and cellular functions category were cell‐to‐cell signaling and interaction, cellular assembly and organization, and cell death and survival. In addition, within the IPA diseases and disorders category, developmental disorder and hereditary disorder were shared between the AS genes obtained between 4‐ and 28‐month‐old wild‐type skin and between the 35‐day‐old HGPS mice and their wild‐type littermates (compare Table S3 and Table 2). The network analysis of AS genes in 35‐day‐old HGPS and wild‐type mice showed that connective tissue disorder, inflammatory disease, and skeletal and muscular disorders as main associated functions had the highest score (Fig. 4E, Table 2). Interestingly, the network with the fourth‐highest score related RNA post‐transcriptional modification functions to genes involved in cancer, two significant functions obtained for the AS genes between 4‐month‐old and 18‐month‐old wild‐type animals across five tissues (compare Table 2 and Table 1). However, these functions were not present for the AS genes between 4‐month‐old and 28‐month‐old skin (Table S3).

### Differential gene expression increases during physiological aging in skin, muscle, and bone

**Figure 5:**
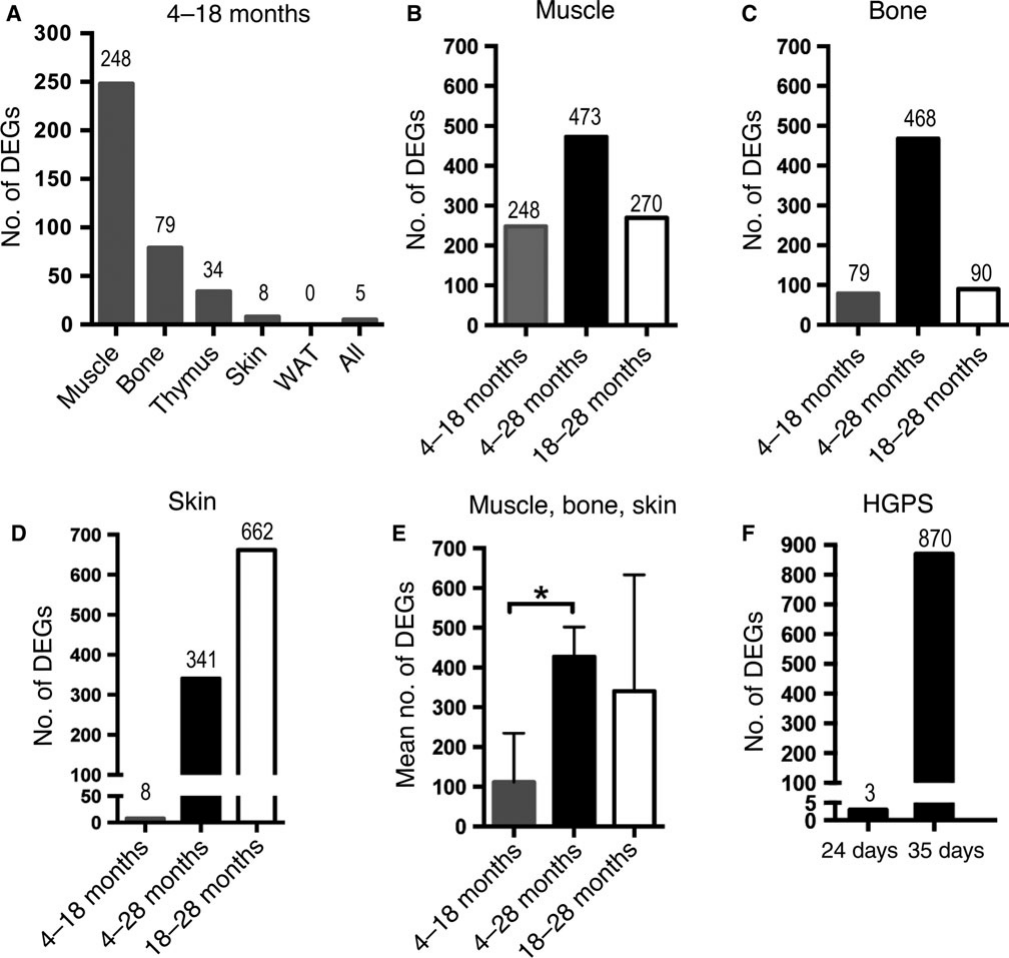
Increased numbers of differentially expressed genes in aging wild‐type and HGPS mice. Number of DE genes (DEGs) with FDR‐adjusted *P*‐value <0.05 and 2‐fold change in gene expression. (A) Distribution of DEGs in the early aging period (4–18 months) by tissue type and across 5 tissues shows a tissue‐specific expression pattern with muscle presenting the highest number of DEGs, but no DEGs in white adipose tissue (WAT). (B) Comparison of DEGs in the muscle between all age groups. (C) Comparison of DEGs in the bone between all age groups. (D) Comparison of DEGs in the skin between all age groups. (E) Mean numbers of DEGs in muscle, bone, and skin show a significant increase in the number of DEGs in the longest aging period (4–28 months) compared to the earlier aging period (4–18 months). (F) Comparison of DEGs between HGPS and wild‐type littermates shows almost no DEGs at postnatal day 24 compared to postnatal day 35. *P <* *0.05: two‐tailed, unpaired t‐test, the data are expressed as the means ± SEM.

To evaluate whether the same pattern of increased number of AS genes associated with age also occurred with the numbers of differentially expressed (DE) genes associated with age, we analyzed the number of DE genes by tissue between the different aging periods (File S3). In the earlier 4‐ to 18‐month aging period of wild‐type mice, we identified 248 genes that were DE in muscle, 79 in bone, 34 in thymus, 8 in skin, and none in white adipose tissue (Fig. 5A). Similar to the increase in the number of AS genes, the number of DE genes increased with advanced age. There were more genes that were DE in the 4‐ to 28‐month aging period than between the 4‐ to 18‐month aging period in muscle, bone, and skin (Fig. 5B–D). The increase in the number of DE genes was significant when considering the three tissues (Fig. 5E). Similar to the levels of AS genes in the skin (in which no genes were found to be alternatively spliced in the 4‐ to 18‐month age period), there were only 8 genes that were found to be DE at the same age period. This was in contrast with the greater number of DE genes detected in the 18‐ to 28‐month aging period in the skin (662 genes) (Fig. 5D). The number of DE genes between 4‐month‐old and 18‐month‐old animals across the five tissues revealed that only five genes were differentially expressed (Fig. 5A, File S3). Given the low number of genes obtained, we chose not to perform enrichment analysis on this data set.

### Differential gene expression increases with age in HGPS

Differential gene expression analysis was also performed in the data set from HGPS and wild‐type littermate controls to investigate whether an increased number of DE genes were observed with increased age in HGPS (File S4). At 24 days of transgenic expression of the HGPS mutation, there were only three genes that were DE between HGPS and wild‐type littermate controls (Fig. 5F). However, after 35 days of expression of the HGPS mutation, the number of genes being differentially expressed had increased to 870 (Fig. 5F, File S4). The gene list of 870 DE genes was analyzed using Webgestalt for GO and KEGG pathway enrichment. The DE genes were enriched for development and cell proliferation functions as well as for metabolic, ECM–receptor interaction, and cytokine–cytokine receptor interaction KEGG pathways (Table S4). Common biological functions with AS genes in HGPS corresponded to the ECM–receptor interaction and focal adhesion, cell death and survival, cellular development, and dermatological disease and conditions (compare Table 2 and Table S4). We also compared enrichment results for DE genes in HGPS to the biological functions obtained for DE genes for skin in normal aging between 4‐month‐old and 28‐month‐old animals (Table S5, File S3), but we did not observe an overlap between shared biological functions (compare Tables S4 and S5).

### Validation of the alternative splicing and gene expression analysis methods

**Figure 6:**
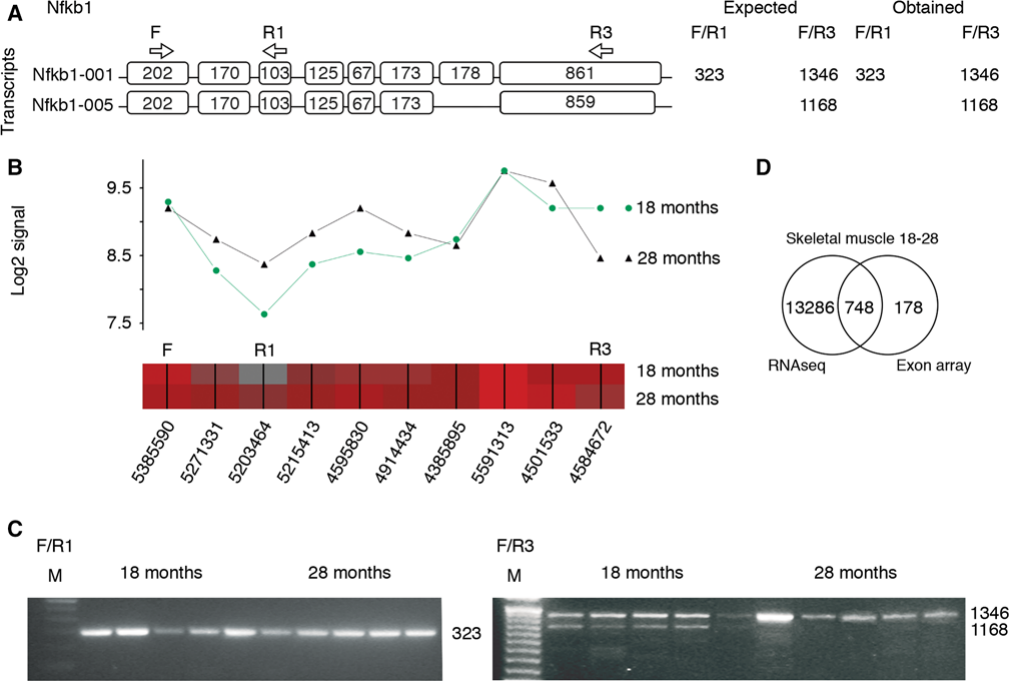
Validation of alternatively spliced genes. (A–C) Alternative splicing of the *Nfkb1* gene detected by Partek's alternative splicing ANOVA at FDR‐adjusted alt‐splice *P*‐value <0.05 is shown as an example. (A) Transcript panel shows Ensembl references for each isoform. Exon sizes in base pair (bp), forward primer (F), reverse primer 1 (R1), R3, and primer positions (arrows) and expected and obtained amplicon sizes (in bp). (B) Overlay of the expected *Nfkb1* transcript isoforms with Partek gene view containing the means of the probe set log2 signals for 18‐ (*n* = 5) and 28‐month‐old (*n* = 5) wild‐type mice (top panel) and probe set heat map with probe set IDs (lower panel, red (high) gray (low) expression). (C) RT–PCR validation shows AS in the putative region of *Nfkb1*. A 323‐bp product is observed in the skin of 18‐ and 28‐month‐old animals using primers F and R1 (left panel). The presence of alternative splicing in the skin of 18‐month‐old animals with two amplification products (using primers F and R3) compared to a single amplification product in the skin of 28‐month‐old animals using the same primers (right panel). M, 100 bp (Invitrogen). (D) Venn diagram of overlapping AS genes between different platforms (RNA sequencing and exon arrays) in the skeletal muscle of 18‐ and 28‐month‐old wild‐type mice, which shows a considerable overlap, validating 80.8% of the AS genes obtained by exon arrays.

Validation of alternative splicing analysis was performed by RT–PCR on the skin from 18‐ and 28‐month‐old mice. Ten genes were selected of the top 17 AS genes from the obtained gene list from pairwise comparison between the skin at 18 and 28 months (File S1). Nine of the 10 genes showed amplification products of the expected sizes by RT–PCR, indicating that these assays were able to detect transcripts (Table S6). Furthermore, six of the nine genes, corresponding to 66.7%, revealed RT–PCR results with the presence of more than one transcript isoform, indicative of alternative exon usage within the putative spliced region (Fig. 6 and Table S6).

A second approach for the validation of alternative splicing included alternative splicing analysis of data generated by RNA sequencing of the skeletal muscle from 18‐ and 28‐month‐old mice (File S5). The RNA sequencing results showed 47 992 exon features being significant after FDR correction at the cutoff of 0.05. These exon features were found in 14 034 unique genes. Comparison of genes detected by exon arrays and RNA sequencing identified 748 genes that were shared between the two platforms (Fig. 6D). This gave a validation of 80.8% of the AS genes from the exon arrays. Network analysis using Wikipathway analysis showed that mRNA processing was the top affected pathway for AS of the skeletal muscle from 18‐ and 28‐month‐old mice using both platforms (Tables S7 and S8).

Changes in gene expression were validated by quantitative RT–PCR (q‐PCR) in the keratinocytes from 35‐day‐old HGPS and control mice. For the analysis of gene expression from the exon arrays, the criteria were defined following validation by q‐PCR. The results from the q‐PCR analysis validated changes in gene expression at a cutoff of FDR‐adjusted *P‐*value <0.05 in combination with a 2‐fold change in gene expression in all genes analyzed (*n* = 13), and hence, these were the criteria used for gene expression analysis. Differentially expressed genes with FDR‐adjusted *P*‐value <0.05 but with a fold change ranging from 1.2 to 1.9 or an adjusted *P*‐value >0.05 did not validate to the same extent. The results from the q‐PCR have been published previously (Rosengardten *et al*., 2011).

## Discussion

In this study, we have investigated the effect of aging on alternative exon usage of the entire genome and applied Partek's alternative splicing ANOVA to determine, in several tissues, the numbers of AS genes between different age groups. Our results showed that the number of alternatively spliced genes increases with age. This trend is evident in both wild‐type mice during physiological aging and in a premature aging mouse model, suggesting that alternative splicing might be more important in the aging process than has previously been noted. Our results are in agreement with those of other recent studies that have shown an effect of age on AS in human brain (Tollervey *et al*., 2011; Mazin *et al*., 2013), human peripheral blood leukocytes (Harries *et al*., 2011), and senescent fibroblasts induced by telomere shortening (Cao *et al*., 2011). To our knowledge, this is the first study to show that the amount of age‐related AS increases with more advanced age (4–18 months compared to 4–28 months) and in several tissues.

Our results showed that the number of alternatively spliced genes varies between different tissues and time periods of the physiological aging process. The most dramatic increase was present in skin, where AS seemed to accelerate in the later aging period (18–28 months), in contrast to in bone where most of the AS change occurred over the whole aging period (4–28 months) with only a few genes in the later aging period (18–28 months). We speculate that these results might reflect a regulatory mechanism that makes splice‐prone tissues less sensitive to the aging‐induced effects from increased number of AS genes. This might also be connected to the fact that AS changes in a large number of genes are related to the appearance, development, and maintenance of cancer.

The total number of AS genes during the entire 4‐ to 28‐month period varied from 690 to 1247 genes between tissues (for skeletal muscle, bone, and skin). This was represented by a range between 13.1–19.1% of the total number of the genes analyzed. This result can be compared to that of the Mazin *et al*.'s (2013) study in human brain using high‐throughput sequencing, which found that nearly 40% of the genes expressed in human brain had changes in splicing over postnatal life. Of these, 70% occurred during development and 30% during aging, and 12% of the total number of genes expressed in the brain had aging‐related splicing (Mazin *et al*., 2013). However, when we considered all compared aging periods (and the five tissues), these numbers varied from 0 to 1886 AS genes and were represented by a range between 0 and 30.4% of the total number of genes that were analyzed, which suggests a considerable variability in the number of AS genes between tissues and ages.

The difference between different tissues when it comes to number of genes being alternatively spliced, with skin having no alternatively spliced genes at 4–18 months, while other tissues such as the adipose tissue and skeletal muscle having more than 100 alternatively spliced genes, might be related to the turnover of the tissue, a process that is slowed by aging. Skin has a high turnover, and as it is constantly regenerated and undergoes high levels of mechanical stress, it might need to be more resistant to impairment. However, adipose tissue and skeletal muscle are mainly composed of postmitotic cells and have a much lower turnover, so the need for stringency is of less importance. But with increased age and slowed tissue regeneration, alternative splicing becomes more common also for the skin. In addition, this variation among different tissues could also be related to tissue‐specific modification of expression levels of transcription factors and splicing factors that could have either a direct or indirect effect on pre‐mRNA splicing (Meshorer & Soreq, 2002).

Surprisingly, we found that the age‐related subset of AS genes across all tissues corresponded to genes involved in RNA post‐transcriptional processing, including genes involved in RNA processing and the spliceosome pathway. At the same time, the most significant group within diseases and disorders with AS genes was cancer. The involvement of AS in cancer has been widely recognized (Singh & Cooper, 2012). Moreover, our results showed that during normal aging, several components of the mouse spliceosome pathway were affected, meaning that the splicing machinery would in particular be affected by age‐related AS, which is in agreement with another *in vivo* study that showed in human blood an effect of age on RNA processing (Harries *et al*., 2011). However, AS genes in senescent fibroblasts induced by telomere shortening have been reported to be involved in remodeling of the cytoskeleton, but not in RNA processing (Cao *et al*., 2011). This disagreement might be related to the differences in the origin of the cells (Gaidatzis *et al*., 2009). Our study, together with Harries *et al*. (2011), suggests that RNA processing, including RNA splicing, is altered during normal physiological aging in both mice and humans. Our results directly implicate AS of genes coding for important components of the spliceosome such as spliceosome proteins (Wbp11 and Prp43) but also snRNAs, involved in spliceosomal catalytic competence (Brow, 2002; Guo *et al*., 2009), which could have implications in splicing specificity and catalysis of specific genes. Furthermore, explorative network analysis identified AS genes involved in RNA post‐transcriptional modifications in direct relation with histone H3 and RNA polymerase II and the NF‐κB complex as a major upstream and central node. Interestingly, the IKK/NF‐κB signaling pathway has been proposed to be one of the key mediators of aging (Huang *et al*., 2003; Wu *et al*., 2006).

When analyzing keratinocytes from HGPS mice and their wild‐type littermates for differences in the numbers of genes with AS, the number of AS genes was lower in HGPS mice (between 24 and 35 days when compared to wild‐type between the same time points), suggesting that the expression of the HGPS mutation had an inhibitory effect on normal developmental splicing. Previous results showed that at postnatal day 24, the skin was in the anagen or growth phase and at postnatal day 35, the skin was in the catagen or regression phase (Hanif *et al*., 2009). However, when we compared the number of genes that were AS between HGPS and wild‐type mice at postnatal day 35, we found that more genes were AS after 35 days, compared to after 24 days of postnatal transgenic expression. Even though additional analysis is needed, this finding might indicate that the expression of the HGPS mutation resulted in increased numbers of genes being alternatively spliced, which suggests a shared mechanism of increased alternative splicing with normal aging. Further studies are needed on different tissues and at different developmental stages to draw a final conclusion about the role of progerin in AS of genes during the development of HGPS.

Gene enrichment analysis of AS genes in HGPS did not reveal post‐transcriptional processing within the main affected functions, as it was found in normal aging mice, but was included within one of the top‐4 AS gene networks, relating these genes to other network‐associated functions such as cancer and gene expression. Cao *et al*. (2011) showed that telomere shortening induced extensive AS and increased progerin production in normal senescent cells. However, they did not show evidence that AS was directly induced by progerin, while our results showed that sustained progerin expression is associated with increased AS in HGPS.

AS genes in HGPS were overrepresented in relevant functions such as skin development, ECM–receptor interaction pathway, and connective tissue disorders. AS genes in HGPS also included genes involved in inflammatory diseases. This finding is in agreement with previous studies that have found an upregulated expression of inflammatory genes in fibroblasts from HGPS patients and keratinocytes from HGPS mice (Adler *et al*., 2007; Rosengardten *et al*., 2011; McKenna *et al*., 2014).

Similar to the increase in the number of AS genes, the number of DE genes increased with advanced age in wild‐type skin, skeletal muscle, and bone. This was also evident in keratinocytes from the HGPS mice at 35 days of age, which showed 870 genes to be differentially expressed at this time point compared to the 24‐day time point, when only three genes were DE. Our results might suggest that during the aging process, increased alternative splicing goes hand in hand with an increased differential gene expression. These processes are likely not independent, but instead one can imagine that there is a set of genes that contributes to both of these programs. This in turn would reflect the normal aging pathology, because differences in the aging‐related degenerative changes were more prominent between 4‐ and 28‐month‐old animals than between 4‐ and 18‐month‐old animals.

Consistent with previous studies, our results show enrichment of the ECM–receptor interaction pathway with DE genes in our HGPS mouse model after 35 days of transgenic expression, but more importantly, our study provides additional evidence for alterations of the ECM components by AS that would alter its composition. ECM components have previously been described to be the second‐largest group of genes misregulated in HGPS cells (Csoka *et al*., 2004). Furthermore, DE genes in 35‐day‐old HGPS mice were involved in system development, cellular proliferation, NF‐κB signaling pathway, and also the cytokine–cytokine receptor interaction pathway. The latter pathway has important roles in the inflammatory response and defense, cell growth, differentiation, and cell death and was also implicated in our study by AS genes in HGPS and by DE genes of normal aging mice as a common denominator.

In summary, our results showed that during the aging process, the number of genes being AS increases with the increased age of the skin, muscle, and bone. To our knowledge, this study is one of the first to describe AS during physiological aging in several tissues, suggesting a shared mechanism of increased AS with increased age which might affect RNA processing, such as the splicing machinery. Our results highlight the importance of global transcriptome studies to advance our understanding of the genetic mechanisms of the aging process.

## Experimental procedure

### Experimental animals

Mice were housed in the experimental animal facility with a 12‐h light/dark cycle, a temperature of 20–22 °C, and 50–75% air humidity (Karolinska Hospital, Huddinge, Sweden). The animal studies were approved by the Stockholm South Ethical Review Board, Dnr S101‐12, S107‐09, and S141‐06. Gene Expression Omnibus numbers accompanies the paper GSE67289, GSE67287, GSE67288 and GSE74274.

## Author contributions

SAR, DG, and ME conceived and designed the study. SAR, DG, and TM performed the experiments. SAR, TM, BB, NST, and MRE performed and/or oversaw the bioinformatic analysis. SAR, DG, TM, MRE, and ME analyzed the data. SAR, TM, and ME wrote the manuscript. All authors read and approved the final manuscript.

## Funding

This study was supported by grants from the Swedish Research Council. This work was also supported in part by the Intramural Research Program of the National Human Genome Research Program, National Institutes of Health.

## Conflict of interest

The authors declare that no conflict of interests exist.

## Supporting information


**Table S1.** GO cellular component enrichment analysis of alternative spliced genes between 4 and 18 months old animals across five tissues.

**Table S2.** GO cellular component enrichment analysis of alternative spliced genes between 35 days old HGPS mice and their wild‐type littermates.

**Table S3.** Enrichment analysis of alternative spliced genes between skin from 4‐months and 28‐months old wild‐type mice.

**Table S4.** Enrichment analysis of differentially expressed genes between keratinocytes from 35‐days old HGPS mice and their wild‐type littermates.

**Table S5.** Enrichment analysis of differentially expressed genes between skin from 4‐months and 28‐months old wild‐type mice.

**Table S6.** Results from RT‐PCR validation of 10 genes from the top 17 alternative spliced genes in skin between 18 and 28 months.

**Table S7.** Wikipathway analysis of alternative spliced genes detected by exon arrays between skeletal muscle from 18‐months and 28‐months old wild‐type mice.

**Table S8.** Wikipathway analysis of alternative spliced genes detected by RNA sequencing between skeletal muscle from 18‐months and 28‐months old wild‐type mice.

**Data S1**. Experimental procedure

**File S1.** All the gene lists of alternative spliced genes obtained between different age groups and for each tissue and across all tissues.

**File S2.** Gene lists of alternatively spliced genes between different age and genotype groups, HGPS mice and wild‐type litter mates.

**File S3.** Gene lists of differentially expressed genes between different age groups in all 5 tissues studied for the normal aging mice.

**File S4.** Gene lists of differentially expressed genes between HGPS mice an their wild‐type litter mates and between 24‐days and 35‐days old mice.

**File S5.** Alternative splicing analysis of data generated by RNA sequencing of skeletal muscle from 18‐ and 28‐month‐old mice.
