# Supplementary material for: Global genome splicing analysis reveals an increased number of alternatively spliced genes with aging
Source: Aging Cell. 2015 Dec 21;15(2):267–78. doi: 10.1111/acel.12433 (PMC4783335; doi:10.1111/acel.12433)
Supplement: Supplementary file 1 — Table S1. GO cellular component enrichment analysis of alternative spliced genes between 4 and 18 months old animals across five tissues. [file ACEL-15-267-s001.docx]

| Category | *Adj. p* value | No. of genes | % of genes on list* |  |
| --- | --- | --- | --- | --- |
| GO Cellular Component ^WG^ |  |  |  |  |
| Intracellular | 6.15E -07 | 119 | 75.3 | |
| - Intracellular part | 3.71E -07 | 118 | 74.7 | |
| - Organelle | 1.08E -07 | 110 | 69.6 | |
| - Intracellular organelle | 1.08E -07 | 110 | 69.6 | |
| - Membrane bounded organelle | 1.08E -07 | 102 | 64.6 | |
| - Intracellular membrane bounded organelle | 1.08E -07 | 102 | 64.6 | |
| - Nucleus | 3.03E -09 | 79 | 50.0 | |
| Macromolecular complex | 1.08E -07 | 58 | 36.7 | |
| - Spliceosomal complex | 1.40E -06 | 10 | 6.3 | |
| - Catalytical step 2 spliceosome | 1.55E -06 | 8 | 5.1 | |
|  |  |  |  | |

Table S1. GO cellular component enrichment analysis of alternative spliced genes between 4 and 18 months old animals across five tissues.

*Number of genes on list = 158, ^WG^ Enrichment analysis performed with WebGestalt, heriarquical organization of top-10 GO functions. – Subcategory.
