# Supplementary material for: Global genome splicing analysis reveals an increased number of alternatively spliced genes with aging
Source: Aging Cell. 2015 Dec 21;15(2):267–78. doi: 10.1111/acel.12433 (PMC4783335; doi:10.1111/acel.12433)
Supplement: Supplementary file 2 — Table S2. GO cellular component enrichment analysis of alternative spliced genes between 35 days old HGPS mice and their wild‐type littermates. [file ACEL-15-267-s002.docx]

| Category | *p* value | No. of genes | % of genes on list* |
| --- | --- | --- | --- |
| GO Cellular Component ^WG^ | ***adj. p* value** | **No. of genes** |  |
| Cell | 8.41E -06 | 286 | 75.9 |
| - Cell part | 8.41E -06 | 286 | 75.9 |
| - Intracellular | 2.00E -04 | 249 | 66.0 |
| - Intracellular part | 3.00E -04 | 244 | 64.7 |
| Extracellular matrix | 9.92E -05 | 22 | 6.1 |
| - Extracellular matrix part | 8.77E -05 | 15 | 4.0 |
| - Proteinaceous extracellular matrix | 7.24E -05 | 21 | 5.6 |
| - Collagen | 8.41E -06 | 12 | 3.2 |
| - Fibrillar collagen | 8.41E -06 | 6 | 1.6 |
| - Collagen type V | 2.00E -04 | 3 | 0.8 |
|  |  |  |  |

Table S2. GO cellular component enrichment analysis of alternative spliced genes between 35 days old HGPS mice and their wild-type littermates.

^*^Number of genes on list = 377, ^WG^ Enrichment analysis performed with WebGestalt, hierarchical organization of top-10 GO functions. – Subcategory.
