# Supplementary material for: Global genome splicing analysis reveals an increased number of alternatively spliced genes with aging
Source: Aging Cell. 2015 Dec 21;15(2):267–78. doi: 10.1111/acel.12433 (PMC4783335; doi:10.1111/acel.12433)
Supplement: Supplementary file 3 — Table S3. Enrichment analysis of alternative spliced genes between skin from 4‐months and 28‐months old wild‐type mice. [file ACEL-15-267-s003.docx]

| Category | *p* value | No. of genes | % of genes on list* |  |
| --- | --- | --- | --- | --- |
| GO Biological Processes ^WG^ | ***adj. p* value** |  |  |  |
| Cellular metabolic process | 7.90E-03 | 488 | 42.3 | |
| - Cellular macromolecule metabolic process | 1.00E-02 | 375 | 32.5 | |
| Protein transport | 1.00E-02 | 91 | 7.9 | |
| Macromolecule localization | 1.14E-02 | 127 | 11.0 | |
| - Protein localization | 1.14E-02 | 112 | 9.7 | |
| KEGG Pathways ^WG^ | ***adj. p* value** |  |  | |
| No significant | - | - | - | |
| Canonical Pathways ^IPA^ | ***p* value** |  |  | |
| Tryptophan Degradation X (Mammalian via Tryptamine) | 2.35E-03 | 5 | 0.4 | |
| Putrescine Degradation III | 2.35E-03 | 5 | 0.4 | |
| IL3-3 Signaling | 3.18E-03 | 11 | 1.0 | |
| ErbB signaling | 4.93E-03 | 12 | 1.0 | |
| Thrombopoietin Signaling | 5.29E-03 | 9 | 0.8 | |
| Molecular and Cellular Functions ^IPA^ | ***p* value** |  |  | |
| Cell Death and Survival | 1.54E-05 – 3.46E-02 | 165 | 14.3 | |
| Cellular Compromise | 2.08E-04 – 3.46E-02 | 45 | 3.9 | |
| Cell-To-Cell Signaling and Interaction | 2.78E-04 – 3.46E-02 | 86 | 7.5 | |
| Cellular Assembly and Organization | 4.65E-04 – 3.46E-02 | 222 | 19.3 | |
| Cellular Function and Maintainance | 4.65E-04 – 3.46E-02 | 214 | 18.6 | |
| Diseases and Disorders^IPA^ | ***p* value** |  |  | |
| Cancer | 2.46E-07 – 3.46E-02 | 904 | 78.4 | |
| Developmental Disorder | 7.31E-05 – 3.46E-02 | 38 | 3.3 | |
| Hereditary Disorder | 7.31E-05 – 3.46E-02 | 112 | 9.7 | |
| Metabolic Disease | 7.31E-05 – 2.31E-02 | 16 | 1.4 | |
| Neurological Disease | 2.08E-04 – 3.46E-02 | 94 | 8.2 | |
| Networks ^IPA^  and Associated Network Functions | **score** |  |  | |
| Hematological System Development and Function, Hematopoiesis, Hereditary Disorder | 46 | 33 | 2.9 | |
| Dermatological Diseases and Conditions, Infectious Disease, Nervous system development | 45 | 35 | 3.0 | |
| Carbohydrate Metabolism, Connective Tissue Disorders, Developmental disorder | 43 | 32 | 2.8 |  |
| Lipid Metabolism, Small Molecule Biochemistry, Developmental Disorder | 41 | 31 | 2.7 |  |
| Developmental Disorder, Hereditary Disorder, Neurological Disease | 41 | 31 | 2.7 |  |
|  |  |  |  |  |

Table S3. Enrichment analysis of alternative spliced genes between skin from 4-months and 28-months old wild-type mice.

*Number of genes on list = 1153, ^WG^ Enrichment analysis performed with WebGestalt, - Subcategory, ^IPA^ Enrichment analysis performed with Ingenuity Pathway Analysis.
