# Supplementary material for: Global genome splicing analysis reveals an increased number of alternatively spliced genes with aging
Source: Aging Cell. 2015 Dec 21;15(2):267–78. doi: 10.1111/acel.12433 (PMC4783335; doi:10.1111/acel.12433)
Supplement: Supplementary file 4 — Table S4. Enrichment analysis of differentially expressed genes between keratinocytes from 35‐days old HGPS mice and their wild‐type littermates. [file ACEL-15-267-s004.docx]

| Category | *p* value / Score | No. of genes | | % of genes on list* |  |
| --- | --- | --- | --- | --- | --- |
| GO Biological Processes ^WG^ | ***adj. p* value** |  |  | |  |
| System development | 1.71E-15 | 222 | 25.5 | | |
| Cell proliferation | 5.87E-15 | 124 | 14.3 | | |
| Response to chemical stimulus | 6.63E-15 | 164 | 18.9 | | |
| Regulation of cell proliferation | 1.64E-14 | 107 | 12.3 | | |
| Organ Development | 4.99E-14 | 173 | 19.9 | | |
| KEGG Pathways ^WG^ | ***adj. p value*** |  |  | | |
| Metabolic pathways | 8.93E-05 | 85 | 9.8 | | |
| ECM-receptor interaction | 3.00E-04 | 15 | 1.7 | | |
| Cytokine.cytokine receptor interaction | 3.00E-04 | 27 | 3.1 | | |
| Focal adhesion | 2.30E-03 | 22 | 2.5 | | |
| Hematopopietic cell lineage | 2.30E-03 | 13 | 1.5 | | |
| Canonical Pathways ^IPA^ | ***p* value** |  |  | | |
| LPS/IL -1 mediated inhibition of RXR function | 4.02E-08 | 29 | 3.3 | | |
| Granulocyte adhesion and diapedesis | 1.92E-07 | 24 | 2.8 | | |
| NF-kB signaling | 7.38E-07 | 24 | 2.8 | | |
| Hepatic fibrosis/ Hepatic stellate cell activation | 8.40E-07 | 26 | 3.0 | | |
| Agranulocyte adhesion and diapedesis | 1.96E-02 | 23 | 2.6 | | |
| Molecular and Cellular Functions ^IPA^ | ***p* value** |  |  | | |
| Cellular growth and proliferation | 3.64E-22 – 2.45E-05 | 356 | 40.9 | | |
| Cellular movement | 9.59E-20 – 2.43E-05 | 239 | 27.5 | | |
| Cell death and survival | 2.28E-18 – 2.55E-05 | 325 | 37.4 | | |
| Cellular development | 3.42E-18 – 2.65E-05 | 336 | 38.6 | | |
| Cell morphology | 2.26E-12 – 1.21E-05 | 239 | 27.5 | | |
| Diseases and Disorders^IPA^ | ***p* value** |  |  | | |
| Cancer | 5.12E-23 – 2.62E-05 | 707 | 81.3 | | |
| Dermatological disease and conditions | 3.98E-22 – 1.59E-05 | 150 | 17.2 | | |
| Inflammatory response | 4.60E-17 – 1.92E-05 | 223 | 25.6 | | |
| Inflammatory disease | 2.23E-14 – 2.23E-05 | 173 | 19.9 | | |
| Cardiovascular disease | 7.70E-14 – 2.56E-05 | 155 | 17.8 | | |
| Networks ^IPA^  and Associated Network Functions | **Score** |  |  | | |
| Cellular growth and proliferation, Tissue development, Cellular movement | 41 | 30 | 3.4 | | |
| Tissue development, Cellular development, Cellular growth and proliferation | 41 | 30 | 3.4 | | |
| Lipid metabolism, Small molecule biochemistry, Cellular assembly and organization | 35 | 27 | 3.1 | |  |
| Lipid metabolism, Small molecule biochemistry, Drug metabolism | 35 | 27 | 3.1 | |  |
| Cell death and survival, Cellular assembly and organization, Cellular compromise | 35 | 27 | 3.1 | |  |
|  |  |  |  | |  |

Table S4. Enrichment analysis of differentially expressed genes between keratinocytes from 35-days old HGPS mice and their wild-type littermates.

*****Number of genes on list = 870, ^WG^ Enrichment analysis performed with WebGestalt, - Subcategory, ^IPA^ Enrichment analysis performed with Ingenuity Pathway Analysis, - Subcategory.
