# Supplementary material for: Global genome splicing analysis reveals an increased number of alternatively spliced genes with aging
Source: Aging Cell. 2015 Dec 21;15(2):267–78. doi: 10.1111/acel.12433 (PMC4783335; doi:10.1111/acel.12433)
Supplement: Supplementary file 5 — Table S5. Enrichment analysis of differentially expressed genes between skin from 4‐months and 28‐months old wild‐type mice. [file ACEL-15-267-s005.docx]

| Category | *P* value / score | No. of genes | % of genes on list* |  |
| --- | --- | --- | --- | --- |
| GO Biological Processes ^WG^ | ***adj. P* value** |  |  |  |
| No Significant | - | - | - | |
| KEGG Pathways ^WG^ | ***adj. P* value** |  |  | |
| Circadian rhythm - mammal | 6.00E-04 | 2 | 5 | |
| Canonical Pathways ^IPA^ | ***P* value** |  |  | |
| Proline Biosynthesis I | 8.91E-03 | 1 | 2.5 | |
| Proline Biosynthesis II (from Arginine) | 1.33E-02 | 1 | 2.5 | |
| Arginine Degradation VI (Arginase II Pathway) | 1.33E-02 | 1 | 2.5 | |
| Glutathione Redox Reactions I | 3.52E-02 | 1 | 2.5 | |
| TCA Cycle II (Eukaryotic) | 4.81E-02 | 1 | 2.5 | |
| Molecular and Cellular Functions ^IPA^ | ***P* value** |  |  | |
| DNA Replication, Recombination, and Repair | 7.25E-05 – 2.65E-02 | 3 | 7.5 | |
| Energy Production | 7.25E-05 – 1.33E-02 | 3 | 7.5 | |
| Nucleic Acid Metabolism | 7.25E-05 – 4.47E-03 | 3 | 7.5 | |
| Small Molecule Biochemistry | 7.25E-05 – 4.38E-02 | 9 | 22.5 | |
| Cell Signaling | 1.73E-04 – 1.73E-04 | 2 | 5.0 | |
| Diseases and Disorders^IPA^ | ***P* value** |  |  | |
| Connective Tissue Disorders | 3.07E-04 – 4.38E-02 | 15 | 37.5 | |
| Metabolic Disease | 3.07E-04 – 2.61E-03 | 7 | 17.5 | |
| Skeletal and Muscular Disorders | 3.07E-04 – 4.38E-02 | 15 | 37.5 | |
| Developmental Disorder | 6.48E-04 – 4.38E-02 | 10 | 25.0 | |
| Hereditary Disorder | 6.48E-04 – 6.69E-03 | 9 | 22.5 | |
| Networks ^IPA^  and Associated Network Functions | **score** |  |  | |
| Connective tissue development and function, Skeletal and muscular system development and function, Tissue Morphology | 32 | 14 | 35.0 | |
| Cardiovascular system development and function, Organismal development, Tissue morphology | 29 | 13 | 32.5 | |
| Cardiovascular disease, Cancer, Endocrine system disorders | 24 | 11 | 27.5 |  |
| Cell death and survival, Cellular function and maintenance, Hematological system development | 3 | 1 | 2.5 |  |
|  |  |  |  |  |

Table S5. Enrichment analysis of differentially expressed genes between skin from 4-months and 28-months old wild-type mice.

*Number of genes on list = 40, ^WG^ Enrichment analysis performed with WebGestalt, - Subcategory, ^IPA^ Enrichment analysis performed with Ingenuity Pathway Analysis, - Subcategory.
