# Supplementary material for: Global genome splicing analysis reveals an increased number of alternatively spliced genes with aging
Source: Aging Cell. 2015 Dec 21;15(2):267–78. doi: 10.1111/acel.12433 (PMC4783335; doi:10.1111/acel.12433)
Supplement: Supplementary file 6 — Table S6. Results from RT‐PCR validation of 10 genes from the top 17 alternative spliced genes in skin between 18 and 28 months. [file ACEL-15-267-s006.doc]

Table S6. Results from RT-PCR validation of 10 genes from the top 17 alternative spliced genes in skin between 18 and 28 months.

|  |  |  |  |  |  |  |
| --- | --- | --- | --- | --- | --- | --- |
| **Gene symbol** | **Forward primer** | **Reverse primer** | **Expected fragment size (bp)** | **Obtained fragment size at 18 months (bp)** | **Obtained fragment size at 28 months (bp)** | **Comment** |
| *Snrnp200* | Snrnp200_F | Snrnp200_R1 | 330, 356 | 330 | 330 | Reference splicing |
|  | Snrnp200_F | Snrnp200_R2 | 591 | 591 | 591 | Reference splicing |
| *Iqgap* | Iqgap1_F | Iqgap1_R1 | 693 | 693 | 693 | Reference splicing |
|  | Iqgap1_F | Iqgap1_R2 | 488 | 488 | 488 | Alternative splicing |
| *Cd163* | Cd163_F | Cd163_R1 | 1199 | 900, 600, 350, 250 | 900, 600, 350, 250 | Alternative splicing |
|  | Cd163_F | Cd163_R2 | 191 | 191, 500 | 191, 500 | Alternative splicing |
| *Nfkb1* | Nfkb1_F | Nfkb1_R1 | 323 | 323 | 323 | Reference splicing |
|  | Nfkb1_F | Nfkb1_R2 | 815, 748 | 815 | 815 | Reference splicing |
|  | Nfkb1_F | Nfkb1_R3 | 1346, 1168 | 1346, 1168 | 1346 | Alternative splicing |
| *Oprm1* | Oprm1_F | Oprm1_R1 | 430 | *-* | *-* | No PCR product |
|  | Oprm1_F | Oprm1_R2 | 245 | *-* | *-* | No PCR product |
| *Rap1gds1* | Rap1gds1_F | Rap1gds1_R1 | 322 | 322 | 322 | Reference splicing |
|  | Rap1gds1_F | Rap1gds1_R2 | 219 | 219 | 219 | Reference splicing |
| *Ccpg1* | Ccpg1_F | Ccpg1_R1 | 536 | 536, 350 | 536, 350 | Alternative splicing |
|  | Ccpg1_F | Ccpg1_R2 | 132 | 132 | 132 | Reference splicing |
| *Podn* | Podn_F | Podn_R1 | 339 | 339, 150 | 339, 150 | Alternative splicing |
|  | Podn_F | Podn_R2 | 1413 | 1413 | 1413 | Reference splicing |
| *Bms1* | Bms1_F | Bms1_R1 | 934 | 934 | 934 | Reference splicing |
|  | Bms1_F | Bms1_R2 | 1363 | - | - | No PCR product |
| *Cyhr1* | Cyhr1_F | Cyhr1_R1 | 365 | 365, 300 | 365, 300 | Alternative splicing |
|  | Cyhr1_F | Cyhr1_R2 | 174 | 174 | 174 | Reference splicing |
|  |  |  |  |  |  |  |

Two genes (*Oprm1* and *Bms1*) failed RT-PCR. Shaded in grey indicate the six genes that validated for alternative splicing by RT-PCR (*Iqgap,Cd163, Nfkb1, Ccpg1, Podn* and *Cyhr1*). Expected fragment size was according to primer BLAST. Obtained fragment size was estimated from agarose gel. Primer sequences are available upon request. Please note that additional transcripts might be available in the databases.
