# Supplementary material for: Global genome splicing analysis reveals an increased number of alternatively spliced genes with aging
Source: Aging Cell. 2015 Dec 21;15(2):267–78. doi: 10.1111/acel.12433 (PMC4783335; doi:10.1111/acel.12433)
Supplement: Supplementary file 7 — Table S7. Wikipathway analysis of alternative spliced genes detected by exon arrays between skeletal muscle from 18‐months and 28‐months old wild‐type mice. [file ACEL-15-267-s007.doc]

Table S7. Wikipathway analysis of alternative spliced genes detected by exon arrays between skeletal muscle from 18-months and

28-months old wild-type mice.

| **Pathway name** | **#Gene** | **Gene symbols** | **Statistics** |
| --- | --- | --- | --- |
| mRNA processing | 41 | *Prpf40a, Mak16, Eif4g2, Xrn2, Dazl, Fxr1, Raly, Ilf3, Afg3l2, Rbm43, Sf3a1, Eif3b, Stau1, Prmt2, Ltv1, Grsf1, Eif4e3, Exosc9, Srpk2, Eral1, Cstf3, Sf3b1, Pcbp1, Hnrnpl, Tial1, Acin1, Puf60, Poldip3, Zcrb1, Clk4, Srrm1, Oas2, Clk3, Rbm6, Prpf3, Ddx25, Dhx9, Rpl8, Cnot4, Hnrnpu, Brwd1* | C=483;O=41;E=7.65;R=5.36;rawP=5.07e-18;adjP=4.97e-16 |
| PluriNetWork | 29 | *Sin3a, Hcfc1, Ctr9, Dazl, Sf1, Rif1, Smad1, Mdm2, Dnmt1, Hif1a, Rel, Hck, Smarcad1, Tle4, Chd4, Paf1, Suz12, Smad4, Ehmt1, Rnf2, Satb2, Grsf1, Dhx9, Trim24, Apc, Rcn2, Hnrnpu, Cdc73, Nr5a2* | C=292;O=29;E=4.63;R=6.27;rawP=6.69e-15;adjP=3.28e-13 |
| EGFR1 Signaling Pathway | 18 | *Sin3a, Pik3cb, Cdc42, Map3k4, Mapk14, Pik3ca, Prkar1a, Stat5b, Hat1, Gja1, Pld1, Ptpn12, Plcg2, Itch, Appl2, Rps6ka5, Grb10, Appl1* | C=217;O=18;E=3.44;R=5.23;rawP=1.53e-08;adjP=5.00e-07 |
| Insulin Signaling | 15 | *Cap1, Sorbs1, Pik3cb, Pdpk1, Rps6kb2, Map3k4, Lipe, Mapk14, Stxbp3a, Rps6ka5, Rhoq, Grb10, Kif3a, Pik3ca, Mapk12* | C=158;O=15;E=2.50;R=5.99;rawP=4.02e-08;adjP=9.85e-07 |
| Myometrial Relaxation and Contraction Pathways | 12 | *Gja1, Plcd1, Rgs14, Gnas, Plcg2, Camk2g, Acta2, Plcb3, Pde4d, Grk6, Prkar1a, Rgs20* | C=158;O=12;E=2.50;R=4.79;rawP=9.32e-06;adjP=0.0002 |
| TNF-alpha NF-kB Signaling Pathway | 14 | *Hsp90ab1, Dap, Smarce1, Tab2, Trpc4ap, Gtf2i, Traip, Chuk, Rpl8, Rps6ka5, Cops3, Rel, Rnf216, Tank* | C=215;O=14;E=3.41;R=4.11;rawP=1.02e-05;adjP=0.0002 |
| Androgen Receptor Signaling Pathway | 10 | *Ncoa2, Sin3a, Smad4, Rac3, Patz1, Rb1, Mdm2, Gtf2f1, Appl1, Ghr* | C=126;O=10;E=2.00;R=5.01;rawP=3.51e-05;adjP=0.0005 |
| Chemokine signaling pathway | 12 | *Cxcl15, Pik3cb, Cdc42, Ccr2, Plcb3, Prkx, Chuk, Grk6, Hck, Pik3ca, Ccl20, Stat5b* | C=186;O=12;E=2.95;R=4.07;rawP=4.72e-05;adjP=0.0006 |
| PPAR signaling pathway | 8 | *Fabp6, Cyp27a1, Rxrg, Sorbs1, Pdpk1, Acox1, Ppard, Ehhadh* | C=93;O=8;E=1.47;R=5.43;rawP=0.0001;adjP=0.0008 |
| TCA Cycle | 5 | *Pdhx, Dlst, Idh3a, Ogdh, Sdhb* | C=32;O=5;E=0.51;R=9.86;rawP=0.0001;adjP=0.0008 |

C, number of genes in the category; O, number of obtained genes in the category; E, number of expected genes in the category; R, ratio of enrichment;

rawP, *P* value from hypergeometric test; adj *P,* *P* value adjusted by the multiple test adjustment
