# Supplementary material for: Global genome splicing analysis reveals an increased number of alternatively spliced genes with aging
Source: Aging Cell. 2015 Dec 21;15(2):267–78. doi: 10.1111/acel.12433 (PMC4783335; doi:10.1111/acel.12433)
Supplement: Supplementary file 8 — Table S8. Wikipathway analysis of alternative spliced genes detected by RNA sequencing between skeletal muscle from 18‐months and 28‐months old wild‐type mice. [file ACEL-15-267-s008.doc]

Table S8. Wikipathway analysis of alternative spliced genes detected by RNA sequencing between skeletal muscle from 18-months and

28-months old wild-type mice.

| **Pathway name** | **#Gene** | **Gene symbols** | **Statistics** |  |
| --- | --- | --- | --- | --- |
| mRNA processing | 357 | *Tdrd7, Ppp1r8, Sf3b3, Clk3, Slc6a8, Ddx1, Snrpd2, Sf3b4, Celf4, Srsf9, U2af1, Rnpepl1, Exosc4, Srsf1, Aco1, Afg3l1, Akap1, Slc25a4, Hnrnpd, Auh, Bard1, Brca1, Cirbp, Clk1, Clk2, Col4a3, Cpeb1, Celf1, Dazl, Dhx15, Ddx3x, Ddx6, Dhx9, Ddx19a, Eif4e, Aimp1, Celf2, Ewsr1, Igf2bp3, Srsf10, Fbl, Ppm1g, Fxr1, Hnrnpc, Hnrnpa1, Hnrnpab, Hnrnpk, Hnrnpl, Prmt1, Trmt2a, Elavl1, Ilf3, Uhmk1, Eif2d, Rbm39, Ppargc1b, Myef2, Zfp638, Npm1, Pabpc1, Abcb4, Papola, Ppp1r14b, Ppargc1a, Eif2ak2, Prpf4b, Ptbp1, Dicer1, Prpf8, Eif4a3, Slu7, Qk, Rad21, Rbm3, Rbm6, Rbmx, Rbmxl1, Rbpms, Rnps1, Rpl22, Mrpl23, Rpl26, Rpl32, Rpl7, Rpl9, Polr2a, Rps14, Rps24, Rps29, Rps4x, Rps6, Rps7, Sf3a2, Atxn1, Srsf2, Srsf3, Srsf5, Tra2b, Slbp, Smn1, Eftud2, Snrpc, Snrnp70, Snrpb, Snrpb2, Snrpd1, Snrpe, Son, Rbms3, Spop, Esrp1, Srp14, Srpk1, Srpk2, Trim21, Trove2, Ssb, Nhp2l1, Stau1, Rsrc2, Dis3l2, Cpeb3, Rbmx2, Enox2, Supt5, Synj2, Dnd1, Tarbp2, Rnpep, Papolg, Alyref, Dhx8, Srp68, Tnrc6c, Tia1, Tial1, Srek1, Traf6, Tsn, Zrsr2, U2af2, Wbp4, Eif4h, Safb2, Srsf7, Ybx1, Cstf3, Csde1, Ptbp3, Pabpc4, Eif4g3, Tardbp, Grsf1, Sfswap, Elmod3, Fus, Sugp2, Ddx19b, Lingo1, Rbm10, Rbm41, Fxr2, Enox1, Oas1g, Oasl2, Rnasel, Rngtt, Smc1a, Srp54a, Xrn2, Rbm45, Pskh1, Rbm47, Csad, Oas3, Oas2, Oas1a, Rbfox1, Cpsf7, Zfp346, Rpl8, Eif4e2, Srp9, Rps11, Ddx24, Nufip1, Nelfe, Lsm2, Eif3b, Imp4, Exosc5, Zfp385a, Adat1, Sf3b2, Zmat4, Vprbp, Akap17b, Ltv1, Slirp, Rbm33, Gatc, Lsm4, Exosc9, Hnrnpdl, Cpsf2, Srrm1, Rbm34, Ddx56, Zfp622, D19Bwg1357e, Nxf1, Eif3g, Hnrnpa2b1, Snrpa, Clasrp, Nono, Cnot4, Tlr5, Sart3, Ddx20, Rps28, Cpsf4, Cpsf3, Prpf40b, Ppie, Rbm38, Prpf40a, Ptbp2, Ddx21, Acin1, Hnrnph2, Ggcx, Mettl3, Spen, Adar, Rbms2, Mbnl1, Rbms1, Rps27, Srsf4, Park7, Eral1, Zbp1, Hnrnph1, Pcbp4, Pcbp3, Rbm8a, Dhx38, Sf3b6, Lsm7, Nip7, 1110037F02Rik, Mrps28, Srp19, Mrpl11, Exosc7, Rps23, Zmat2, Snrnp40, Rdm1, Pspc1, Sltm, Rae1, Rbm4b, Rbm22, Rbm7, Rpl11, Rbm25, Ttc14, Zcrb1, Lsm1, Prpf18, Rpl39, Rpl37, Snrpd3, Cstf1, Rnmtl1, Ppil4, Ankrd33b, Ilkap, Ergic2, Sf3a1, Cpeb4, Rpl38, Polr2g, Nop9, Rbm18, Rnmt, Mak16, Wdr55, Nifk, U2surp, Puf60, Srsf6, Snrpg, Rps13, Ncbp2, Fdx1l, Rpl39l, Rnaset2b, Nudt21, Rbm28, Ddx39, Ciz1, Phf5a, Tmed10, Dnajc8, Zfp740, Prpf6, Snrpa1, Dhx16, Srsf11, Dnajc17, Afg3l2, Exosc8, Rnaseh2a, Snrpf, Tut1, Prpf4, Cd2bp2, Dazap1, Taf15, Sugp1, Prpf3, Rbm43, Cdc40, Rbpms2, Cyp4f18, Lsm11, Rbm12b1, Htatsf1, Bclaf1, Dis3, Hnrnpll, Ddx41, Poldip3, Rbm19, Hnrnpr, Sf3a3, Secisbp2, Snrnp35, Lsm8, Msi2, Rps9, Rbm17, Rbm12b2, Pum1, Pum2, Sf3b1, Rbm5, Bicc1, Rbfox2, Brwd1, Cpsf1, Trmt1l, Hnrnpf, Clp1* | C=483;O=357;E=107.19;R=3.33;rawP=3.59e-130;adjP=6.46e-128 |  |
| PluriNetWork | 230 | *Gadd45gip1, Etv5, Cabin1, Kdm3a, Rcor2, Ogt, Ehmt2, Raf1, Acvr1, Acvr1b, Akt1, Mta1, Apc, Nr2f2, Axin1, Bmp4, Bmpr2, Brca1, Klf5, Casp3, Ctnnb1, Ccnd1, Cd44, Cdh1, Cdk2, Cdkn1a, Cdkn2a, Creb1, Crebbp, Dpysl2, Ctbp1, Ctcf, Dgka, Dazl, Ddb1, Gadd45a, Dhx9, Dffa, Dnmt1, Dnmt3a, Dnmt3b, Cdk2ap1, Dvl1, Lefty1, Phc1, Eed, Nr2f6, Nr2f1, Smarcad1, Ewsr1, Ezh1, Ezh2, Fgf5, Fgfr1, Fos, Gab1, Gata6, Gdf9, Grb2, Aes, H3f3a, Hcfc1, Hck, Hdac2, Hells, Hif1a, Hras, Icam1, Id1, Igfbp3, Il6st, Kpnb1, Inhbb, Irs1, Itgb1, Jak1, Jarid2, Klf2, Klf4, Kpna2, Lif, Lifr, Lrp5, Lyar, Smad1, Smad2, Smad3, Smad4, Smad7, Mbd2, Mbd3, Mdm2, Mef2c, Mef2d, Mitf, Pias2, Mtf2, Myc, Myod1, Ncoa1, Nfkb1, Mycn, Nodal, Notch1, Uhrf1, Npr1, Ocln, P4ha1, Pik3cd, Pim1, Prkaca, Pml, Psen1, Pten, Ptpn11, Ptprs, Rbbp4, Rbl2, Rbpj, Rel, Rela, Relb, Rest, Rnf2, Rock1, Rock2, Rras, Satb1, Sgk1, Shh, Sin3a, Smarcc1, Kdm5c, Sos1, Sp1, Sp3, Spp1, Ssrp1, Zfp143, Stat3, Hdac4, Tfe3, Satb2, Tbx3, Tcf7, Tcf7l1, Tfeb, Cdc73, Socs2, Kdm6b, Terf2, Tgfb1, Tgfbr1, Trim24, Trim28, Tle2, Tle4, Nr2c1, Trp53, Ctr9, Twist1, Ube2i, Sumo1, Kdm6a, Pim3, Wnt5a, Atrx, Ercc5, Yy1, Ipo9, Sf1, Zfp57, Zfx, Gatad2b, Grsf1, Ipo7, Gatad2a, Leo1, Mta2, Pin1, Rbbp7, Usp7, Esrrb, Map2k1, Mapk1, Mapk3, Nr5a2, Rcn2, Acvr1c, Jade1, Smo, Kmt2d, Hdac1, Ppp2r1a, Rif1, Tet1, Suz12, Dnmt3l, Paf1, Rybp, Arid3b, Gsk3b, Mtor, Bcam, Pias4, Fam129a, Perp, Wdr61, Nacc1, Wwp2, Pbrm1, Smarca2, Cad, Zfp219, Stk40, Yipf2, Smurf1, Zmym2, Plet1, Kdm4c, Cers2, Ehmt1, Elp4, Kat5, Nedd4l, Smarca5, Nkd1, Trim33, Sall4, Kdm1a* | C=292;O=230;E=64.80;R=3.55;rawP=4.30e-94;adjP=3.87e-92 | |
| Focal Adhesion | 168 | *Itga9, Pelo, Mylk, Rapgef1, Actn1, Braf, Rap1a, Raf1, Actb, Akt1, Akt2, Birc3, Birc2, Araf, Rhoa, Arhgap5, Bad, Bcl2, Blk, Capn1, Cav1, Cav2, Cav3, Ccnd1, Ccnd2, Ccnd3, Cdc42, Chad, Col11a1, Col11a2, Col3a1, Col4a1, Col4a2, Col4a4, Col5a1, Col5a2, Col6a2, Col1a1, Col1a2, Bcar1, Crk, Crkl, Diap1, Egf, Egfr, Erbb2, Ptk2, Fgr, Figf, Flt1, Fn1, Fyn, Grb2, Hck, Hgf, Igf1, Ilk, Itga2b, Itga3, Itga4, Itga5, Itga6, Itga7, Itgae, Itgal, Itgam, Itgav, Itgax, Itgb1, Itgb2, Itgb3, Itgb5, Itgb6, Itgb7, Lama2, Lama3, Lama4, Lama5, Lamb1, Lamb2, Lamb3, Lamc2, Parvb, Rac3, Met, Myl6, Ppp1r12a, Pak1, Pak3, Pdgfa, Pdgfb, Pdgfrb, Pdpk1, Pgf, Pik3ca, Pik3cd, Pik3r1, Pik3r2, Pip5k1c, Pten, Flna, Itgb4, Pxn, Rac1, Rac2, Reln, Rock1, Rock2, Sepp1, Shc1, Sos1, Spp1, Src, Srms, Rap1b, Thbs1, Thbs2, Thbs3, Thbs4, Tln1, Tnc, Txk, Vasp, Vav1, Vcl, Vegfa, Vegfb, Vegfc, Vtn, Vwf, Pak2, Mapk4, Lamc1, Farp2, Zyx, Mylk2, Akt3, Lamc3, Map2k5, Mapk7, Itga8, Pak7, Styk1, Map2k1, Map2k2, Map2k3, Map2k6, Mapk1, Mapk8, Mapk9, Mapk12, Pik3cg, Itga11, Pik3r5, Itgb8, Dock1, Mapk6, Tnk2, Col5a3, Pdgfc, Gsk3b, Pak4, Pdgfd, Pik3cb, Pik3r4, Tnxb, Tnk1, Col4a6* | C=186;O=168;E=41.28;R=4.07;rawP=3.49e-88;adjP=2.09e-86 | |
| Insulin Signaling | 144 | *Rhoq, Prkaa1, Rapgef1, Prkaa2, Raf1, Rps6ka3, Akt1, Akt2, Arf1, Cap1, Cbl, Socs3, Crk, Egr1, Ehd1, Eif4e, Eif4ebp1, Flot1, Flot2, Fos, Gab1, Grb10, Grb2, Gys1, Hras, Igf1r, Ikbkb, Inppl1, Irs1, Irs3, Kif3a, Kif5b, Lipe, Myo1c, Enpp1, Pdpk1, Pfkl, Pfkm, Pik3c2a, Pik3ca, Pik3cd, Pik3r1, Pik3r2, Pik3r3, Prkca, Prkcb, Prkcd, Prkch, Prkci, Prkcq, Prkcz, Ppp1cc, Mapk11, Cyth3, Pten, Ptpn1, Ptpn11, Ptprf, Rab4a, Rac1, Rac2, Rheb, Rps6ka1, Rps6ka2, Sgk1, Sorbs1, Shc1, Slc2a1, Slc2a4, Snap25, Snap23, Sos1, Sos2, Srf, Cblb, Stx4a, Stxbp1, Stxbp2, Stxbp3, Stxbp4, Tbc1d4, Shc2, Tsc2, Vamp2, Xbp1, Map4k3, Pik3c3, Mapk4, Trib3, Gys2, Arhgap33, Sh2b2, Map2k5, Mapk7, Ehd2, Map2k1, Map2k2, Map2k3, Map2k4, Map2k6, Map3k1, Map3k11, Map3k12, Map3k2, Map3k3, Map3k4, Map3k5, Map3k7, Map3k8, Map4k1, Map4k2, Mapk1, Mapk10, Mapk14, Mapk3, Mapk8, Mapk9, Inpp4a, Map4k4, Map3k10, Gyg, Mapk12, Pik3cg, Map3k9, Map4k5, Mapk6, Grb14, Mink1, Map3k6, Rrad, Foxo1, Foxo3, Rps6ka4, Gsk3b, Mtor, Rps6kb2, Tsc1, Rps6ka6, Map3k13, Rps6kb1, Rps6ka5, Pik3cb, Pik3r4, Rhoj* | C=158;O=144;E=35.06;R=4.11;rawP=4.64e-77;adjP=2.09e-75 | |
| TNF-alpha NF-kB Signaling Pathway | 167 | *Fbxw11, Rnf216, Txlna, Alpl, Akt1, Akt2, Birc3, Birc2, Bcl3, Btrc, Casp2, Casp3, Casp7, Casp8, Cav1, Cdc37, Cflar, Chuk, Cradd, Crebbp, Csnk2a1, Csnk2a2, Csnk2b, Ddx3x, Mark2, Fadd, Ptk2, Faf1, Fbl, Fkbp5, Gab1, Gtf2i, Hdac2, Hdac6, Hspb1, Hsp90ab1, Hsp90aa1, Ikbkb, Ikbkg, Kcnq1, Kpna2, Kpna3, Kpna6, Ktn1, Mcm5, Psmd7, Nfkb1, Nfkb2, Nfkbia, Nfkbib, Nfkbie, Nsmaf, Pdcd2, Peg3, Pfdn2, Prkaca, Prkcz, Papola, Pml, Psmc1, Psmc2, Psmc3, Eif4a3, Flna, Ptpn11, Ripk2, Rel, Rela, Relb, Dpf2, Rpl6, Polr1c, Polr1b, Polr1d, Polr1a, Glg1, Smarcb1, Smarcc1, Src, Tifa, Fancd2, Tank, Skp1a, Cdc34, Ccar2, Tnf, Tnfaip3, Tnfrsf11a, Tnfrsf1a, Tnfrsf1b, Tnfrsf8, Nr2c2, Traf1, Traf2, Traf3, Traf4, Traf6, Traip, Psmd3, Ube2i, Sumo1, Dap, Ywhae, Ywhag, Ywhah, Ywhaz, Rasal2, Zfand5, Pias3, Ikbkap, Tnip2, Usp11, G3bp2, Map2k5, Pebp1, Psmd13, Polr2h, Map3k1, Map3k2, Map3k3, Map3k8, Cops3, Casp8ap2, Rpl8, Cul1, Rps11, Azi2, Pkn1, Mcc, Ppp1r13l, Hdac1, Usp2, Ywhab, Iqgap2, Akap8, Trpc4ap, Actl6a, Tbk1, Ikbke, Ripk3, Gsk3b, Smarce1, Rnf25, Tnip1, Polr1e, Psmd6, Tab1, Tab3, Psmd12, Bag4, Rpl4, Rps13, Smarcc2, Tab2, Nkiras1, Cd3eap, Tradd, Dcaf7, Nkiras2, Lrpprc, Rps6kb1, Rps6ka5, Cyld, Mtif2, Bcl7a, Nfkbiz,* | C=215;O=167;E=47.71;R=3.50;rawP=5.12e-67;adjP=1.84e-65 | |
| Chemokine signaling pathway | 148 | *Ccl27a, CR974586.2, Gm13306, Adcy4, Adcy3, Arrb1, Braf, Rap1a, Raf1, Adrbk1, Adcy6, Adcy7, Adcy9, Akt1, Akt2, Rhoa, Cxcr5, Cdc42, Chuk, Cxcr2, Cxcr3, Cxcr4, Ccr1, Ccr2, Ccr5, Bcar1, Crk, Crkl, Csk, Cx3cr1, Elmo1, Ptk2, Fgr, Gnai1, Gnai2, Gnai3, Gnb1, Gnb3, Gnb4, Gnb5, Gng12, Gng2, Gng3, Gng5, Gng7, Gng8, Gngt2, Grk4, Grk5, Grb2, Cxcl1, Hck, Hras, Cxcl10, Ikbkb, Ikbkg, Itk, Jak2, Kras, Xcl1, Lyn, Cxcl9, Ncf1, Nfkb1, Nfkbia, Nfkbib, Nras, Pak1, Pik3ca, Pik3cd, Pik3r1, Pik3r2, Pik3r3, Prkaca, Prkacb, Prkcb, Prkcd, Prkcz, Plcb1, Plcb2, Plcb3, Plcb4, Ccl21a, Prkx, Ptk2b, Pxn, Rac1, Rac2, Rasgrp2, Rela, Rock1, Rock2, Ccl11, Ccl12, Ccl2, Ccl22, Ccl25, Ccl3, Ccl4, Ccl5, Ccl6, Ccl7, Ccl8, Ccl9, Cxcl2, Cx3cl1, Cxcl12, Shc1, Sos1, Sos2, Stat2, Stat3, Stat5b, Adcy2, Rap1b, Shc2, Tiam1, Vav1, Vav2, Was, Adcy5, Akt3, Tiam2, Grk6, Map2k1, Mapk1, Mapk3, Shc4, Prex1, Pik3cg, Pik3r5, Adcy1, Cxcl13, Ccl24, Foxo3, Gsk3b, Pf4, Ccl28, Vav3, Cxcl14, Ppbp, Gsk3a, Gng11, Cxcl16, Wasl, Pik3cb, Cxcr6, Pard3, Dock2* | C=186;O=148;E=41.28;R=3.59;rawP=4.39e-62;adjP=1.32e-60 | |
| MAPK signaling pathway | 136 | *Gck, Arrb1, Braf, Rap1a, Raf1, Rps6ka3, Rasa2, Acvr1b, Akt1, Akt2, Atf4, Bdnf, Casp1, Casp2, Casp3, Casp6, Casp7, Casp8, Casp9, Cd14, Cdc25b, Cdc42, Crk, Crkl, Daxx, Gadd45a, Ddit3, Egf, Egfr, Fas, Fos, Gna12, Grb2, Hspa5, Nr4a1, Hspb1, Ikbkb, Ikbkg, Il1b, Il1r1, Il1r2, Kras, Tmem37, Mapkapk2, Max, Mef2c, Mras, Mapt, Myc, Nf1, Nfkb1, Ngf, Nlk, Nras, Pak1, Pdgfb, Pdgfrb, Prkaca, Prkcb, Prkcd, Prkch, Prkcz, Pla2g5, Ppm1a, Ppm1b, Ppp3ca, Ppp3cb, Ppp3cc, Ppp5c, Flna, Dusp1, Ptprr, Rac1, Rac2, Rasgrp1, Sos2, Srf, Rap1b, Taok1, Tgfb1, Tgfb2, Tgfb3, Tgfbr1, Tgfbr2, Rasa1, Tnf, Traf2, Traf6, Trp53, Pak2, Map4k3, Mapk4, Rasgrp4, Dusp7, Akt3, Map2k5, Mapk7, Dusp5, Map2k1, Map2k2, Map2k4, Map2k6, Map3k1, Map3k11, Map3k12, Map3k4, Map3k5, Map3k7, Map3k8, Map4k1, Mapk1, Mapk10, Mapk14, Mapk3, Mapk8, Mapk9, Map4k4, Acvr1c, Ecsit, Mapk12, Dusp4, Ptpn7, Taok2, Mapk6, Mink1, Map3k6, Stk3, Dusp10, Zak, Tab1, Dusp6, Tab2, Hspb2, Map3k13, Ntf5* | C=165;O=136;E=36.62;R=3.71;rawP=9.16e-61;adjP=2.36e-59 | |
| Myometrial Relaxation and Contraction Pathways | 127 | *Adcy4, Adcy3, Dgkz, Atf5, Camk2d, Cald1, Arrb1, Acta1, Actb, Actc1, Acta2, Adcy6, Adcy7, Adcy9, Adm, Gpr182, Atf1, Atf3, Atf4, Atp2a2, Cacnb3, Calca, Calm1, Calm2, Calm3, Camk2a, Camk2b, Camk2g, Ackr3, Cnn1, Cnn2, Crcp, Creb1, Creb3, Atf6b, Fos, Gabpa, Gabpb1, Gja1, Gnaq, Gnas, Gnb1, Gnb3, Gnb4, Gnb5, Gng12, Gng2, Gng3, Gng5, Gng7, Gng8, Lpar1, Grk4, Grk5, Gsto1, Igfbp3, Igfbp4, Igfbp5, Igfbp6, Il1b, Il6, Itpr1, Itpr2, Maff, Myl4, Myl2, Nfkb1, Nos1, Nos3, Oxtr, Pde4b, Prkaca, Prkacb, Prkca, Prkcb, Prkcd, Prkce, Prkch, Prkci, Prkd1, Prkcq, Prkcz, Pkia, Pkib, Pkig, Plcb3, Plcd1, Plcg1, Prkar1a, Prkar2a, Prkar2b, Rgs16, Rgs2, Rgs4, Rgs5, Ryr1, Ryr3, Slc8a1, Sp1, Adcy2, Adcy5, Ywhae, Ywhag, Ywhah, Ywhaz, Mylk2, Plcg2, Ets2, Pde4d, Rgs7, Grk6, Adcy1, Rgs1, Rgs6, Rgs3, Rgs11, Rgs14, Ramp1, Atp2a3, Corin, Ywhab, Ramp3, Rgs19, Rgs17, Rgs18, Gng11, Rgs10* | C=158;O=127;E=35.06;R=3.62;rawP=2.05e-54;adjP=4.61e-53 | |
| EGFR1 Signaling Pathway | 154 | *Hat1, Snrpd2, Pkn2, Raf1, Krt7, Rps6ka3, Abi1, Akt1, Ap2a1, Araf, Arf4, Atf1, Camk2a, Casp9, Cav1, Cav2, Cbl, Cdc42, Socs3, Creb1, Bcar1, Crk, Crkl, Csk, Asap1, Dnm1, Dok2, Egf, Egfr, Elf3, Epn1, Eps15, Eps15l1, Eps8, Fos, Gab1, Gab2, Gja1, Grb10, Grb2, Grb7, Ptpn6, Htt, Hras, Inppl1, Itch, Jak1, Jak2, Kras, Krt18, Krt8, Smad2, Smad3, Myc, Nck1, Nck2, Nras, Pak1, Pik3ca, Pik3cd, Pik3r1, Pik3r2, Pik3r3, Pitpna, Prkca, Prkcb, Prkci, Prkd1, Prkcz, Plcg1, Pld1, Pld2, Prkar1a, Ptk2b, Ptpn11, Ptpn12, Dusp1, Ptprr, Pxn, Rac1, Reps2, Klf11, Reps1, Rfxank, Ralgds, Rgs16, Ralbp1, Rps6ka1, Rps6ka2, Sh3gl2, Shc1, Sin3a, Snca, Sos1, Sos2, Sp1, Src, Stat2, Stat3, Stat5a, Stat5b, Cblb, Stxbp1, Hip1, Appl2, Git1, Tgif1, Rasa1, Plscr1, Vav1, Vav2, Zpr1, Wnk1, Plcg2, Map2k5, Mapk7, Mta2, Pebp1, Spry2, Pik3c2b, Rbbp7, Ceacam1, Map2k1, Map2k2, Map2k3, Map3k1, Map3k2, Map3k3, Map3k4, Mapk1, Mapk14, Mapk3, Mapk8, Rab5a, Sh2d3c, Pik3cg, Hdac1, Grb14, Tnk2, Ywhab, Shoc2, Foxo1, Vav3, Tnip1, Sh3kbp1, Ralb, Ndufa13, Yjefn3, Appl1, Rps6ka5, Wasl, Errfi1, Pik3cb, Usp6nl* | C=217;O=154;E=48.16;R=3.20;rawP=6.47e-53;adjP=1.29e-51 | |
| T Cell Receptor Signaling Pathway | 117 | *Prkd2, Cabin1, Rapgef1, Card11, Braf, Rap1a, Abi1, Abl1, Acp1, Akt1, Arhgdib, Bcl10, Ctnnb1, Cbl, Cd2, Cd2ap, Cd3d, Cd3e, Cd3g, Cd4, Cd8a, Cdc42, Cish, Creb1, Crebbp, Crk, Crkl, Dbnl, Dlg1, Dnm2, Enah, Evl, Ptk2, Fos, Dtx1, Fyn, Gab2, Grb2, Ptpn6, Itk, Itpr1, Lat, Lcp2, Sh2b3, Lyn, Grap2, Muc1, Nck1, Nedd9, Nfatc2, Pak1, Pik3r1, Pik3r2, Prkcq, Plcg1, Ppp3cb, Pstpip1, Ptk2b, Ptpn11, Ptpn12, Ptpn22, Ptprc, Ripk2, Ptprj, Pxn, Rac2, Rasgrp2, Khdrbs1, Shc1, Sla, Sos1, Sos2, Src, Stat5a, Stat5b, Cblb, Syk, Wipf1, Rasa1, Tuba4a, Tubb5, Txk, Unc119, Vasp, Vav1, Vav2, Was, Zap70, Shb, Def6, Mapk7, Sh3bp2, Lax1, Wasf2, Map2k1, Map2k2, Map3k1, Map4k1, Mapk1, Mapk3, Git2, Homer3, Sh2d2a, Sh2d3c, Stk39, Arhgef7, Skap2, Ptpn3, Hdac7, Vav3, Grap, Dusp3, Arhgef6, Nfam1, Skap1, Dock2, Pag1* | C=143;O=117;E=31.74;R=3.69;rawP=8.43e-52;adjP=1.52e-50 | |

C, number of genes in the category; O, number of obtained genes in the category; E, number of expected genes in the category; R, ratio of enrichment; rawP, *P* value from hypergeometric test; adj *P,* *P* value adjusted by the multiple test adjustment
