# Supplementary material for: Global genome splicing analysis reveals an increased number of alternatively spliced genes with aging
Source: Aging Cell. 2015 Dec 21;15(2):267–78. doi: 10.1111/acel.12433 (PMC4783335; doi:10.1111/acel.12433)
Supplement: Supplementary file 9 — Data S1. Experimental procedure [file ACEL-15-267-s009.doc]

**Supporting information;**

**Global genome splicing analysis reveals an increased number of alternatively spliced genes with aging**

Sofía A. Rodríguez, Diana Grochová, Tomás McKenna, Bhavesh Borate, Niraj S. Trivedi, Michael R. Erdos, Maria Eriksson

**EXPERIMENTAL PROCEDURE**

**Experimental animals**

Mice were supplied with R36 pellets (Lactamin, Sweden) and drinking water *ad libitum*. Male C57BL/6J wild-type mice were aged until 4, 18 and 28 months of age and euthanized by cervical dislocation. Five mice were studied for each age group. Ventral skin, skeletal muscle from the thigh, bone from the tibia, abdominal white adipose tissue and thymus were dissected from each mouse at 4 and 18 months of age. In addition, ventral skin, skeletal muscle and bone were also collected from the 28-month-old animals. Extra care was taken during dissection and collection of tissue to ensure that the time ranged between 30 and 45 minutes and that the tissues were immediately transferred to liquid nitrogen. Tissues were stored at -80° C prior to homogenization and RNA extraction.

Keratinocytes were obtained from 24- and 35-day-old HGPS mice, FVB/N.Cg-Tg (tetop-LA^G608G^,-EGFP) VF1-07; K5tTA (Sagelius *et al.,* 2008a), with transgenic expression induced at the day of birth, and from their wild-type littermates (n=4 per individual age and genotype group), as previously described (Rosengardten *et al.,* 2011). All C57BL/6J wild-type mice included in this study were weighted directly after cervical dislocation. Animals with health problems or tumors in their internal anatomy were discarded from the study.

Ventral skin and skeletal muscle from the thigh were dissected from the 3 different age groups (4, 18 and 28 months; n=4 or 5 for each age group and tissue) and preserved overnight in 4% PFA and stored in 70% ethanol. Tissues were embedded in paraffin, sectioned and stained with hematoxylin and eosin for histopathological analysis. Femurs were dissected from three age groups (n=5 per age-group), and the length and mid-diaphysis thickness were measured with calipers in 2 dimensions. Body weights were measured at different time points (4 months, n=14; 18 months, n=6; 28 months, n=5).

**RNA extraction**

RNA was extracted with Trizol reagent (Ambion) from tissues or primary keratinocytes according to the manufacturer’s recommendation. Skeletal muscle, thymus and white adipose tissue were homogenized in Trizol. Liquid nitrogen-frozen bones and ventral skin were homogenized using a protein Mikro Dismembrator S (B. Braun, Biotech International, Melsungen, Deutschland), at 18,000 rpm for 45 seconds, and the powder was thawed in Trizol. Total RNA was further purified using an RNeasy cleanup kit (Qiagen) and quantified using a standard OD spectrophotometer and a NanoDrop spectrophotometer (NanoDrop Technologies, DE, USA). The RNA integrity was assessed by the Agilent 2100 Bioanalyzer (Agilent, CA, USA). The RNA quality criteria for proceeding with exon arrays were a 18S/28S ratio above 1.5 and an RNA integrity number (RIN-value) ≥ 8.0 (in agreement with the recommendations from the Bioinformatics and Expression Analysis core facility at the Karolinska Institutet, Sweden).

**Exon arrays**

The Affymetrix GeneChip® Mouse Exon 1.0 ST is a sense strand array containing 1.2 million probe sets of an average of 4 perfect match probes, with at least one probe set per exon and enables gene expression and alternative splicing analysis on a whole-genome scale. The Core Meta Probe set was used and corresponded to 194,293 probe sets of 16,755 transcript clusters with source transcript annotation input from RefSeq and full-length mRNA GenBank records containing complete CDS information. For array hybridization and Whole Transcript Sense Target Labeling Synthesis, 200 ng of RNA was processed using the Gene Chip WT cDNA Synthesis Kit, Affymetrix P/N 900673 and Gene Chip sample cleanup module, Affymetrix P/N 900371 (Affymetrix, Inc., Santa Clara, CA). A total of 5.5 μg of labeled and fragmented single-stranded cDNA was hybridized to the arrays and incubated in a 45º C oven for 16-18 hours, 60 rpm rotation (Affymetrix Gene Chip Hybridization Oven 640). Arrays were washed and stained using the FS450_0001 fluidic script (Affymetrix Gene Chip Fluidics Station 450), and scanning was performed by Affymetrix Gene Chip scanner 3000 7G. Hybridization and scanning of the arrays was performed at the Karolinska Institute Bioinformatics and Expression Analysis core facility.

**Analysis of exon array data**

The core meta probe set of 16,755 transcript clusters, identified as 16,711 annotated genes, were analyzed in Partek Genomic Suite v.6.6 (Partek Inc., St. Louis, MO, USA) at the exon level to detect alternatively spliced genes and at the gene level to study differential gene expression. The data were imported into Partek, with no pre-filtering applied, and array quality control was performed using both Partek and Affymetrix® Expression Console™ (v 1.1). Affymetrix CEL files with exon array raw data were imported into Partek and filtered to only include the core meta probe set. For the normalization of probe signals, RMA background correction algorithm was selected and adjusted for GC content. A quantile normalization and median polish probe set summarization was used, and normalized probe signals were converted into log 2 scale. Probe sets were linked in Partek to current Affymetrix annotation files (NetAffx, release 33.1 Mm9). Exon array hybridization images were visually inspected in Partek image browser to discard technical artifacts on the chip.

Detection of AS genes between the different age groups was performed with Partek’s splicing ANOVA (Partek, Manual). The Affymetrix exon arrays provide exon expression levels that can be used to analyze differential exon usage, which is AS by definition (Affymetrix white paper). We have used Partek’s GS alternative splicing ANOVA to detect alternatively spliced genes. Partek directly uses the exon signals to detect differences in the expression of exons in the ANOVA. It then declares the presence of relevant alternative splice variants when one or more exons appear to have different expression mean values between the two comparison groups. Anova factors were age and mouse ID, and the splice factor was age. Only non-differentially expressed (non-DE) genes were included for the AS analysis, since DE genes have been reported to yield false positive AS genes with exon arrays (Gaidatzis *et al.,* 2009 and Affymetrix white paper: “ Identifying and Validating Alternative Splicing Events”). Therefore, genes with *P*-value <0.05 for differential gene expression were filtered out. The final gene lists contained AS genes with a cutoff point of FDR adjusted alternative splicing (alt-splice) *P-*value <0.05. A gene list was created for the AS analysis and for each pairwise comparison between different age groups and the number of AS genes compared between different aging periods.

For the analysis of exon array data at the gene level, the data were imported into Partek and the same core meta probe set list, summarization and normalization of probe signals was used (see above). Detection of DE genes was in accordance with the pre-defined exon workflow menu in Partek. Exons were summarized to genes and a 2-way ANOVA was applied. Gene lists with DE genes were further filtered by selecting genes that had at least a 2-fold change in combination with a FDR adjusted *P-*value < 0.05. Lists of DE genes were created in Partek for each pair-wise comparison.

Probe set expression data used for the AS and differential gene expression analysis have been submitted to the public Gene Expression Omnibus (Edgar *et al.,* 2002), numbers GSE67289, GSE67287 and GSE67288.

**Gene enrichment analysis**

Gene lists of AS genes and DE genes were analyzed for gene set enrichment of GO functions and KEGG pathways using Webgestalt (Zhang *et al.,* 2005; Wang *et al.,* 2013) and through the use of IPA (Ingenuity® Systems, [www.ingenuity.com](http://www.ingenuity.com/)). In Webgestalt, we used a hypergeometric statistical test, and as multiple test adjustment of *P*-values, we used Benjamini (1995), and a minimum of 2 genes to obtain the top-10 most significant GO functions or KEGG pathways

**RT-PCR validation of exon arrays**

Among the top 17 AS genes, obtained from the AS analysis of the skin from 18 and 28-month-old mice, 10 genes with AS were selected for validation by RT-PCR. The selection of genes met the following criteria: genes composed of more than 3 and less than 50 exons, an AS region that did not involve the last or first exon of the gene (to allow for primers to be designed on flanking exons of the spliced region and with at least one primer inside one of the spliced exons). An aliquot of the same RNA that was hybridized to the exon array was DNase I treated (RQ1 RNase-free DNase, Promega) and purified with RNeasy minicolumns (Qiagen). cDNA synthesis was performed on 800 ng of total RNA with random hexamers using the SuperScript™ cDNA Synthesis Kit (Life technologies). cDNA samples were diluted in nuclease-free water and stored at −20°C. PCR reactions were performed in a 25 μl reaction volume, including 1-3 μl of cDNA template with Hot Star Taq plus Polymerase (Qiagen). The quality of the cDNA template was determined by amplification of β-actin, and each gene was screened with at least two different primer combinations for all 10 skin cDNA samples (18-month-old mice, n=5 and 28-month-old mice n=5).

Quantitative RT-PCR was performed to validate the gene expression data obtained from the exon arrays. Twenty genes involved in Wnt signaling and inflammation were selected for validation (Rosengardten *et al.,* 2011). Details about the experimental procedure and the results have been included in a previous publication from the laboratory (Rosengardten *et al.,* 2011). Primer sequences are available upon request.

**RNA sequencing**

Aliquots from same total RNA extracts processed for exon microarray were used for high-throughput mRNA strand-specific sequencing with Illumina TruSeq and poly-A selection. Skeletal muscle RNA samples from 18-months (n=1) and 28-months (n=3) old animals were sequenced on HiSeq2500 at a depth of 40.5-65.1 million read pairs in lane with PhiX error rate of 0.39% and an average quality score 35.8-35.9 (92.43-92.77% bases > = Q30). Raw sequencing reads were processed following the Tuxedo protocol (Trapnell *et al.,* 2012). The reads for each biological replicate were first mapped independently to the mouse genome Mm9 with TopHat2 and transcripts assembled with Cufflinks, merged to corresponding annotations with Cuffmerge and expression data quantified with Cuffdiff.

Stranded pair-end reads were mapped to the mouse genome genome build GRCm38 using the program Tophat v. 2.0.4 (Kim *et al*., 2013). Gene counts for exons were extracted from the resulting BAM files using HTseq v. 0.5 using a "flattened" GTF file following the manual of DEXseq (Anders *et al*., 2012; Anders *et al*., 2015). Modelling of exon usage as well as detection of differentially expressed exons were done with the DEXSeq package that uses a negative binomial distribution to estimate the biological variance in the experiment and then fits a generalized linear model to identify exons that are differentially expressod after taking gene expression differences into account (Anders *et al*., 2012). All exons with a change in expression that leads to a *P*-value <0.05 after Benjamin-Hochberg correction for multiple testing were treated as exons with evidence of differential exon usage between the two groups in the RNA-sequencing data. A Fisher test, as implemented in the R package TopGO (Alexa and Rahnenfuhrer 2010), were used to identify gene ontology (GO) categories that were significantly overrepresented in the group of significant genes. RNA sequencing data used for the AS analysis have been submitted to the public Gene Expression Omnibus (Edgar *et al.,* 2002), number GSE74274.

**SUPPLEMENTARY REFERENCES**

Alexa A and Rahnenfuhrer J (2010) topGO: topGO: Enrichment analysis for Gene Ontology. R package version 2.18.0.

Anders S, Reyes A and Huber W (2012) "Detecting differential usage of exons from RNA-seq data. Genome Research, 22, pp. 4025.

Anders S, Pyl PT and Huber W (2015) HTSeq — A Python framework to work with high-throughput sequencing data. Bioinformatics, 15, 31(2):166-9.

Benjamini Y (1995). Controlling the false discovery rate: a practical and powerful approach to multiple testing. (Hochberg Y, ed). *J. Roy. Statist. Soc. Ser. B* 57. No 1, 289-300.

Edgar R, Domrachev M, Lash AE (2002). Gene Expression Omnibus: NCBI gene expression and hybridization array data repository. Nucleic Acids Res. 1;30(1):207-10.

Gaidatzis D, Jacobeit K, Oakeley EJ, Stadler MB (2009). Overestimation of alternative splicing caused by variable probe characteristics in exon arrays. Nucleic Acids Res. 37, e107.

Kim D, Pertea G, Trapnell C, Pimental H, Kelley R and Salzberg SL (2013) TopHat2: accurate alignment of transcriptomes in the presence of insertations, deletions and gene fusions. Genome Biology, 14, R36.

Partek, Manual. Alternative splice analysis of exon data in Partek Genomics Suite

Rosengardten Y, McKenna T, Grochova D, Eriksson M (2011). Stem cell depletion in Hutchinson-Gilford progeria syndrome. Aging Cell. 10, 1011-1020.

Trapnell C, Roberts A, Goff L, Pertea G, Kim D, Kelley DR, Pimentel H, Salzberg SL, Rinn JL, Pachter L (2012). Differential gene and transcript expression analysis of RNA-seq experiments with TopHat and Cufflinks. Nat Protoc. 7, 562-578.

White paper: Identifying and Validating Alternative Splicing Events; An introduction to managing data provided by GeneChipÆ Exon Arrays. Affymetrix Technical Notes.

Zhang B, Kirov S, Snoddy J (2005). WebGestalt: an integrated system for exploring gene sets in various biological contexts. Nucleic Acids Res. 33, W741-748.
